# Ca^2+^-dependent nuclease is involved in DNA degradation during the formation of the secretory cavity by programmed cell death in fruit of *Citrus grandis* ‘Tomentosa’

**DOI:** 10.1093/jxb/eraa199

**Published:** 2020-04-23

**Authors:** Mei Bai, Minjian Liang, Bin Huai, Han Gao, Panpan Tong, Rongxin Shen, Hanjun He, Hong Wu

**Affiliations:** 1 State Key Laboratory for Conservation and Utilization of Subtropical Agro-bioresources, South China Agricultural University, Guangzhou, China; 2 University of Trento, Italy

**Keywords:** Ca^2+^-dependent nuclease, *Citrus*, DNA degradation, programmed cell death, secretory cavity

## Abstract

The secretory cavity is a typical structure in *Citrus* fruit and is formed by schizolysigeny. Previous reports have indicated that programmed cell death (PCD) is involved in the degradation of secretory cavity cells in the fruit, and that the spatio-temporal location of calcium is closely related to nuclear DNA degradation in this process; however, the molecular mechanisms underlying this Ca^2+^ regulation remain largely unknown. Here, we identified *CgCaN* that encodes a Ca^2+^-dependent DNase in the fruit of *Citrus grandis* ‘Tomentosa’, the function of which was studied using calcium ion localization, DNase activity assays, *in situ* hybridization, and protein immunolocalization. The results suggested that the full-length cDNA of *CgCaN* contains an ORF of 1011 bp that encodes a protein 336 amino acids in length with a SNase-like functional domain. CgCaN digests dsDNA at neutral pH in a Ca^2+^-dependent manner. *In situ* hybridization signals of *CgCaN* were particularly distributed in the secretory cavity cells. Ca^2+^ and Ca^2+^-dependent DNases were mainly observed in the condensed chromatin and in the nucleolus. In addition, spatio-temporal expression patterns of *CgCaN* and its protein coincided with the time-points that corresponded to chromatin degradation and nuclear rupture during the PCD in the development of the fruit secretory cavity. Taken together, our results suggest that Ca^2+^-dependent DNases play direct roles in nuclear DNA degradation during the PCD of secretory cavity cells during *Citrus* fruit development. Given the consistency of the expression patterns of genes regulated by calmodulin (CaM) and calcium-dependent protein kinases (CDPK) and the dynamics of calcium accumulation, we speculate that CaM and CDPK proteins might be involved in Ca^2+^ transport from the extracellular walls through the cytoplasm and into the nucleus to activate CgCaN for DNA degradation.

## Teaser

The Ca^2+^-dependent DNase CgCAN plays a direct role in nuclear DNA degradation during the programmed cell death that leads to the formation of secretory cavities in the fruit of *Citrus grandis*.

## Introduction

Programmed cell death (PCD) is a process ubiquitous in plant development that occurs at specific times and in specific regions. PCD serves as the final stage of plant cell differentiation, and it is also the basic mechanism used by plants to resist biological and abiotic stresses in the environment. In the past decade a large number of studies have been conducted on plant PCD (Coll *et al.*, 2011); however, the mechanisms underlying developmental PCD remain poorly understood. Some studies have shown that the mechanisms are similar to those in animal cells, not only in terms of morphological and biochemical changes, but also in terms the regulation involved (Reape and McCabe, 2010). For example, cytochemical and immunocytochemical experiments have demonstrated that caspase 3-like and calcium ions are involved in chromatin agglutination and DNA degradation in the apoptosis of citrus fruit secretory vesicles (Liu *et al.*, 2012; Zheng *et al.*, 2014). Ca^2+^ may play a role in hypersensitive PCD by activating caspase 3-like activity (Zuppini *et al.*, 2005), or it may enter the cytoplasm from the extracellular space, increasing intracellular calcium levels and causing vacuole rupture (Fukuda, 2000). In addition, the increase in Ca^2+^ concentration can directly activate calcium-dependent endonucleases, which can be used for DNA degradation (Aleksandrushkina and Vanyushin, 2009).

One of the most significant features of PCD in plants is the gradual degradation of genomic DNA. Nucleases first degrade genomic DNA to create large fragments (Peitsch *et al.*, 1994), and a process known as ‘DNA laddering’ occurs by which it is broken into fragments that are multiples of ~180 bp in length (Jiang *et al.*, 2008). The search for DNases that degrade DNA has become a focus of research in studies on plant PCD.

Many studies have demonstrated that nucleases are involved in nuclear DNA degradation during plant PCD processes such as endosperm development (Bozhkov *et al.*, 2004), flower organ development (Rogers, 2005; Gu *et al.*, 2011), leaf morphogenesis (Gunawardena, 2008), the hypersensitive response (Tada *et al.*, 2001; Hofius *et al.*, 2007), organ senescence (Stein and Hansen, 1999), leaf senescence (Xu and Hanson, 2000; Farage-Barhom *et al.*, 2008), degradation of the aleurone cell layer (Fath *et al.*, 2000), and vessel differentiation (Fukuda, 2000; Chen *et al.*, 2012). Comparative analyses have shown that the nuclease activity involved in PCD is highly dependent on the plant species, the particular tissue involved, and on the developmental stage, and specific nucleases may be required to degrade genomic DNA in each case. Although nuclease activities during PCD have been detected in some species (Chen *et al.*, 2012), their identification at the molecular level has rarely been performed (Aoyagi *et al.*, 1998; Ito and Fukuda, 2002; Gu *et al.*, 2011; Chen *et al.*, 2012; Guo *et al.*, 2012; Sui *et al.*, 2019).

All the nucleases that have been reported in plant PCD are ion-dependent, and on this basis they can be divided into four categories, namely, Zn^2+^-, Ca^2+^-, Mg^2+^-, and Ca^2+^-and Mg^2+^-dependent (Sugiyama *et al.*, 2000; Ito and Fukuda, 2002). Different nucleases are involved in PCD during different stages of plant development. For example, three Ca^2+^-dependent DNases are involved in the degradation of corn endosperm cells (Young and Gallie, 2000) and a 35-kDa Zn^2+^-dependent nuclease, BEN1, degrades aleurone cells in barley seed (Aoyagi *et al.*, 1998). Domínguez and Cejudo (2006) found that Ca^2+^ or Mg^2+^ activate DNase in nucellar cell extracts of wheat (*Triticum aestivum* ‘Chinese Spring’) during PCD and that they are localized together with a serine protease in the nuclei of nucellar cells. Although there are four types of plant nucleases, only Zn^2+^- and Ca^2+^-dependent nucleases are involved in the degradation of dsDNA (Sugiyama *et al.*, 2000). In addition to the specific ion requirements, Zn^2+^-dependent endonuclease mainly act on RNA and ssDNA when the pH is in the acidic range, while Ca^2+^-dependent endonucleases are optimal for dsDNA at neutral pH (Sugiyama *et al.*, 2000). In addition, the activities of Ca^2+^-dependent endonucleases initially increase in the nuclei of cells undergoing PCD and cause a somewhat limited fragmentation of nuclear DNA; in contrast, PCD induces the accumulation of Zn^2+^-dependent endonucleases in the apoplast or vacuoles. Ultimately, membrane systems are broken down, and the nuclear DNA is exposed to extensive degradation by apoplastic or vacuolar Zn^2+^-dependent endonucleases (Sugiyama *et al.*, 2000).

Secretory cavities are a common structural feature in plants of the Rutaceae, and their origins and developmental patterns are generally classified into three types, namely schizogenous, lysigenous, and schizolysigenous (Liang *et al.*, 2006, 2009; Chen and Wu, 2010). Early studies produced controversial and contradictory results due to technical limitations (Turner *et al.*, 1998). More recently, using methods involving electron microscopy combined with enzyme cytochemistry, Liang *et al.* (2009) found that pectinase and cellulase are involved in the separation and dissolution of the cell wall during the development of the secretory cavity, and it has been demonstrated that secretory cavities in *Citrus* plants are of the schizolysigenous type (Liang *et al.*, 2006, 2009). Subsequently, Chen and Wu (2010) used TUNEL assays to show that PCD is involved in the dissolution of secretory cavity cells. Further studies have demonstrated that both caspase 3-like and Ca^2+^ are involved in the degradation of the nuclear structure and of chromatin during the PCD of secretory cavity cells (Liu *et al.*, 2012; Zheng *et al.*, 2014). Thus, PCD has been confirmed to be involved in the formation of secretory cavities during *Citrus* fruit schizolysigeny (Chen and Wu, 2010; Liu *et al.*, 2012). In addition, Zheng *et al.* (2014) found that the spatio-temporal localization of Ca^2+^ is closely related to nuclear morphological changes, and hence it is presumably involved in regulating the degradation of the nuclear structure and nuclear chromatin during the PCD. However, the mechanism underlying the involvement of Ca^2+^ remains unknown.

Three types of Ca^2+^-dependent endonucleases have been detected in immune reactions in tobacco leaves, namely, NUCI, NUCII, and NUCIII, and they are able to degrade both tobacco genomic DNA and ssDNA (Mittler *et al.*, 1995). Hao *et al.* (2003) reported the existence of a Ca^2+^-dependent endonuclease in female flowers of diapause stamens in *Cucumis sativus*, and a later study indicated that this could be involved in anther-specific DNA damage of female cucumber flowers during the earliest stage of development (Gu *et al.*, 2011). Chen *et al.* (2012) isolated two Ca^2+^-dependent endonuclease genes, *EuCaN1* and *EuCaN2*, from the secondary xylem of *Eucommia ulmoides*, and the proteins they encode were found to be located in the nucleus and to be involved in the degradation of ss- and dsDNA during the differentiation of the secondary xylem cells at neutral pH. Similarly, two Ca^2+^-dependent nuclease genes, *CaN1* and *CaN2*, also exist in the genome of Arabidopsis, and their prokaryotic expression results in the degradation of ss- and dsDNA *in vitro* (Isono *et al.*, 2000; Guo *et al.*, 2012). The CaN1 and CaN2 proteins are modified and located on the cell membrane (Leśniewicz *et al.*, 2012). Recently, CaN2 has been reported to reduce tolerance to salt stress and it can induce cell death under such conditions (Sui *et al.*, 2019).

The activation of a Ca^2+^-dependent endonuclease that degrades DNA directly relies on increased Ca^2+^ concentrations (Aleksandrushkina and Vanyushin, 2009). Ca^2+^ precipitated from the cell walls that subsequently accumulates abnormally in the nuclei is associated with, and presumably involved in, PCD in *Citrus sinensis* (Zheng *et al.*, 2014), although the process by which the Ca ions in the nuclei are recruited remains unclear. There are three types of Ca^2+^-binding proteins in plants, namely calmodulin (CaM) and calmodulin-like (CML) proteins, calcineurin B-like (CBL) proteins, and calcium-dependent protein kinases (CDPKs/CPKs) (McCormack *et al.*, 2005; Batistic and Kudla, 2009; Luan *et al.*, 2009; Asano *et al.*, 2012; Boudsocq and Sheen, 2013). These can bind to Ca^2+^ to signals that regulate plant growth and development, immune responses, and stress responses (Wang *et al.*, 2009). CaM, one of the most widely distributed and most important Ca-binding proteins, has high specificity and affinity for Ca^2+^ (Reddy *et al.*, 2001). The properties of CML are similar to CaM (Dobney *et al.*, 2009), and both can regulate the germination and directional growth of pollen tubes by regulating Ca^2+^ concentrations in the pollen tube (Moutinho *et al.*, 1998; McCormack *et al.*, 2005; [Bibr CIT0069][Bibr CIT0034]). CDPK plays an important role in Ca^2+^-mediated signal transduction (Hamel *et al.*, 2014; Romeis and Herde, 2014; Simeunovic *et al.*, 2016). AtCPK16 located in the plasma membrane can transfer Ca^2+^ between the inside and outside of the plasma membrane in Arabidopsis (Giacometi *et al.*, 2012).

In the present study, we cloned a Ca^2+^-dependent DNase gene, *CgCaN*, and utilized a combination of cytologic techniques including *in situ* hybridization and immunocytochemical localization to confirm its involvement in DNA degradation during the PCD of secretory cavity cells in *Citrus grandis* ‘Tomentosa’. Our results provide insights into the mechanism by which Ca^2+^-dependent DNase is involved in the PCD of *Citrus* fruit secretory cells.

## Materials and methods

### Plant materials and sampling

Flowers and fruits of *Citrus grandis* ‘Tomentosa’ were obtained between March and June 2017 from a 10-year-old tree in the nursery garden of South China Agricultural University, Guangzhou, China (23°10′2″N, 113°21′53″E). Tissue samples containing secretory cavities were collected from the ovary wall and fruit exocarp at 10 different developmental stages (H1–H10; Supplementary Fig. S1 at *JXB* online).

Secretory cavities originate in the wall of the unfertilized ovary. After fertilization, secretory cavities continue to occur and develop as the fruit gradually grows and enlarges. At each stage in the fruit developmental process the ovary wall and the exocarp contain both developed and developing secretory cavities. Observations and statistical analysis indicated that most of the secretory cavities at H1 were at the early stage of the initial cells, most of those at H2 were at the middle stage of the initial cells, most of those at H3 were at the late stage of the initial cells, and those at H4–H10 ranged from the stage of formation of the intercellular lumen to the mature stage.

### Light microscopy and TUNEL assays

Small blocks of tissue (0.5×0.5×1 mm) containing secretory cavities at different stages of development were fixed in a phosphate buffer solution (PBS, pH 7.2) containing 4% paraformaldehyde and 0.5% glutaraldehyde. For anatomical observations, the samples were embedded in Epon 812 resin (SPI Supplies). Sections of 1.5 μm thick were cut using a Leica 2255 microtome, stained with Toluidine Blue O, and observed using Leica DM6 B white-light microscopy equipped with a Leica DFC550 imaging system (He *et al.*, 2018).

For the TUNEL assays to detect fragmentation of DNA, ovary walls and fruit exocarps at different developmental stages were cut into 1×1×1 mm blocks and fixed overnight in 4% paraformaldehyde in PBS at 4 °C. The blocks were dehydrated in an alcohol series (15%, 30%, 45%, 60%, 70%, 80%, 90%, and 100%), treated with xylene until transparent, embedded in paraffin (Sigma), and then cut into 5-μm sections using a Reichert HistoSTAT 820 paraffin slicer. TUNEL assays were conducted using a Promega DeadEnd™ Fluorometric TUNEL System (G3250) according to the manufacturer’s instructions. Slides were then immediately stained with 10 mg ml^–1^ propidium iodide (to detect cell death; Sigma-Aldrich) and mounted using SlowFade Gold antifade reagent (Invitrogen). Samples were observed and photographed under a Leica DM6 B microscope equipped with a Leica DFC550 imaging system (He *et al.*, 2018).

Positive controls were prepared by treating the tissues with DNase I before incubation, and negative controls were prepared by treating the tissues with the incubation buffer without the rTdT enzyme, which is a terminal deoxynucleotidyl transferase that labels the blunt ends of breaks in dsDNA independent of a template (see Supplementary Fig. S7).

### Calcium localization

Potassium pyroantimonate is often used to detect and locate intracellular Ca^2+^ because it reacts with it to form deposits with high electron densities (Tian *et al.* 1998).

Small blocks of tissue (0.5×0.5×1 mm) containing secretory cavities at different stages of development were fixed in 0.1 M KH_2_PO_4_ buffer solution (pH 7.8) with 2.5% paraformaldehyde (v/v) containing freshly added 2% potassium pyroantimonate. The samples were washed five times with buffer (fresh buffered 2% potassium pyroantimonate) for 20 min each, post-fixed for 10 h at 4 °C in 2% (w/v)-buffered OsO_4_ containing 1% potassium pyroantimonate, washed five times in buffer without potassium pyroantimonate for 20 min each, dehydrated in a graded ethanol series (15%, 30%, 45%, 60%, 70%, 80%, 90%, and 100%), infiltrated, and then embedded in Epon 812 resin (SPI Supplies). A Leica EM UC7 microtome was used for cutting sections to a thickness of 70–80 nm. After staining with uranyl acetate and lead citrate, the sections were examined and photographed using a Philips Fei-Tecnai 12 TEM (Zheng *et al.*, 2014). As additional controls, sections were incubated in 200 mM EGTA (pH 7.9) for 1 h (Tian *et al.*, 1998).

### Quantitative real-time PCR analysis of *CgCaN*, *PICBP*, and *CDPK* expression

Samples of 100 mg were taken from ovary walls and fruit exocarps containing secretary cavities at the different developmental stages (H1–H10), and from mature mesocarps without secretary cavities, and total RNA was extracted using a Column Plant RNAout 2.0 kit (TIANDZ) according to the manufacturers’ protocol. The RNA concentration was determined using a Nanodrop 2000 spectrophotometer (ThermoFisher Scientific). First-strand cDNA was generated by reverse transcription from 1 μg of total RNA using a PrimeScript^TM^ RT Reagent Kit with gDNA Eraser (Perfect Real Time) (Takara) in a 20-μl reaction volume. Quantitative real-time PCR (qRT-PCR) was performed using the method described by He *et al.* (2018) with SYBR Pre-mix Ex Taq^TM^ (Takara) with a T100 system (BioRad, USA). Three biological and three technical replicates were used for each experiment.

Expression was examined for the Ca^2+^-dependent DNase gene *CgCaN* of *Citrus grandis* ‘Tomentosa’, the calmodulin (CaM) gene *PICBP*, and the family of Ca^2+^-dependent CDPK genes *CDPK5*, *CDPK5-like*, and *CDPK7* of *Citrus sinensis*. *Actin* was used as the internal reference, and the primers used are listed in Supplementary Table S1.

### Cloning and sequence analysis of *CgCaN* cDNAs

Total RNA and first-strand cDNA were obtained as described above. The published sequence of the Arabidopsis Ca^2+^-dependent DNase *AtCaN1* (https://www.ncbi.nlm.nih.gov) was used to BLAST the genome database of *Citrus sinensis* (http://citrus.hzau.edu.cn/cgi-bin/orange/search) to obtain the candidate gene sequence (*Cs7g30310*). This candidate was then compared with other published plant Ca^2+^-dependent DNase genes (*PtCaN*, *Populus trichocarpa*; *EuCaN*, *Eucommia ulmoides*; *AtCaN*, Arabidopsis; *CsCaN*, *Cucumis sativus*) using the DNAMAN8.0 software for further verification. The BLAST results are shown in Supplementary Fig. S2. To complete the gene cloning, the primers for the ORF sequence were designed based on the candidate gene using the Primer Premier 5.0 software (Supplementary Table S1). The 1011-bp cDNA sequence was obtained and then translated into an amino acid sequence using the DNAMAN 8.0 software (Supplementary Fig. S3). The protein was named CgCaN, based on the cDNA sequence.

Analysis of the deduced amino acid sequence of CgCaN of *Citrus grandis* ‘Tomentosa’ was performed using the ProtParam tool, the TMHMM program, and the SignalP program from ExPASy (http://www.expasy.org). Functional domains were predicted using the NCBI Protein BLAST software (http://www.ncbi.nlm.nih.gov).

For phylogenetic analysis (Chen *et al.*, 2012), CgCaN was used as a reference to search for its homologs in the NCBI database (http://www.ncbi.nlm.nih.gov) using BLASTp with a cut-off of 1×10^–80^. The neighbor-joining method implemented in MEGA version 7.0 was used for phylogenetic analysis and was performed using *p*-distance. The reliability of branches was assessed by 1000 bootstrap replications.

### *In vitro* expression of *CgCaN* in *E. coli* and DNase activity assays

To express CgCaN with the His-tag in *E. coli*, the 1011-bp *CgCaN* cDNA containing a ORF was inserted into the pET-30a vector. The optimal conditions for expression were determined to be 1 mM IPTG incubation at 23 °C for 6 h. A sample of 100 ml of the bacterial liquid was centrifuged at 5000 *g* at 4 °C for 10 min, the bacteria were collected. Then 4 ml of His protein binding buffer was added and ultrasonic treatment was applied for 10 min, followed by centrifugation at 12000 *g* for 10 min at 4°C, after which the supernatant was collected.

For protein purification, the following steps were performed. First, an appropriate amount of resin (100 μl resin for 1 ml bacterial liquid) was taken and mixed with 1 ml His-combined buffer, and then centrifuged at 190 *g* at 4 °C for 2 min. This was repeated three times and the supernatant was removed each time and set aside. Second, the lysate (i.e. the bacterial supernatant described above) was added to the recovered resin and was allowed to combine at 4 °C for 3 h. The samples were then centrifuged at 190 *g* at 4 °C for 3 min, after which the supernatant was removed. The resin was washed with His-combined buffer five times to remove the supernatant, and was eluted at 4 °C in 1 ml of His elution buffer (300 μl imidazole, 700 μl His buffer) for 1 h. The supernatant was then centrifuged to obtain the purified protein.

A total of 1 μg purified protein was mixed with rice T65 genome DNA and BD plasmid DNA in the reaction liquid (with or without 0.0001, 0.001, or 0.1 M CaCl_2_) and after 1 h of incubation at 37 °C detection was performed using a 1% agarose gel. As a negative control, T65 genome DNA and BD plasmid DNA were incubated for 1 h at 37 °C in the same buffer without purified protein (Supplementary Fig. S4).

### Protein extraction and western blot analysis

Ovary walls and exocarps with secretary cavities at different developmental stages and mature mesocarps without secretary cavities were collected. A total of 0.2 g of fresh material was extracted using a Microextraction Kit for Total Plant Protein (TIANDZ), and the same amount of proteins from different developmental stages were separated using 12% (w/v) SDS-page and then blotted onto PVDF membranes (200 mA, 72 min). The membranes were then incubated overnight at 4 °C with either purified anti-CgCaN antibodies (PBST dilution 1:1000, primary antibody) or anti-β-actin (PBST dilution 1:7000; 42 kDa, Wuhan ABclonal Biological Technology Co., Ltd), and then washed three times with PBST for 5 min each. The membranes were then incubated in PBST containing colloidal secondary antibodies (1:6000 v/v; Goat Anti Rabbit IgG, Polyclonal, Wuhan ABclonal Biological Technology Co., Ltd) for 1 h at 24 °C, and washed five times with PBST for 5 min each. This was followed by color development using horseradish peroxidase (HRP)-enhanced chemiluminescence (ECL) assays. The results for anti-β-actin labeled proteins from the different developmental stages are shown in Supplementary Fig. S5A. In the control A, pre-immunization serum was used to replace the primary antibody, and in control B the primary antibody was replaced with PBS (Supplementary Fig. S5).

For the preparation of anti-CgCaN-specific polyclonal antibodies, the full-length protein (336 aa) of the CgCaN coding region was cloned into the pET-28a expression vector and expressed in *E. coli* strain Rosetta (DE3). The target immunogen was purified from inclusion bodies, solubilized in urea, and then mixed with Freund’s adjuvant for injection into two Japanese big-ear rabbits for *in vivo* immunization; this was done at the Wuhan ABclonal Biological Technology Co., Ltd. Rabbits were injected four times over 60 d. The anti-CgCaN serum was collected, and the reactivity to the antigen was determined by ELISA. The antiserum was purified using an affinity column conjugated with purified recombinant CgCaN protein. The results for endogenous proteins (from *in vitro* expression) and exogenous proteins (from the fruit exocarps of *C. grandis* ‘Tomentosa’) are shown in Supplementary Fig. S5D.

### Gene expression analysis by *in situ* hybridization

Small blocks of tissue (1×1×1 mm) containing secretory cavities at different development stages were fixed with 4% paraformaldehyde and embedded in paraffin. Longitudinal sections of 6 μm thickness were mounted on glass slides coated with poly-L-lysine. Transverse and longitudinal sections of 6 μm thickness were hybridized with sense and anti-sense probes. Detection of hybridization was conducted using the following steps: 37 °C pre-heating, proteinase K and acetylated solution treatment, dehydration, overnight probe hybridization, and color development. The labeled signals were analysed using light microscopy (Leica DM6 B).

A 277-bp fragment from positions 659–936 of *CgCaN* was used to generate a probe. The PCR fragment was inserted into the XbaI to HindIII sites of the pGEM-T Easy vector (Promega), and was transcribed *in vitro* from either the T7 or SP6 promoter for either sense or antisense strand synthesis using a Digoxigenin RNA labeling kit (Roche) according to the manufacturer’s instructions.

### Immunocytochemical localization

Small blocks of tissue (0.5×0.5×1 mm) containing secretory cavities at different developmental stages were fixed in a PBS solution (pH 7.2) containing 4% paraformaldehyde and 0.5% glutaraldehyde, and embedded in LR White resin (Sigma). A Leica EM UC7 microtome was used for cutting the sections to a thickness of 70–80 nm.

A nickel grid containing the sections was washed three times with a droplet of PBS-Tween buffer solution for 5 min, and then blocked with 1% BSA for 20 min. The grid was washed three times with PBST for 5 min each and was then floated on PBST containing the anti-CgCaN-specific polyclonal antibodies (primary antibodies, 1:10 v/v) and left to incubate for 3 h at 37 °C. The grid was again washed three times with PBST for 5 min each, then floated on PBST containing colloidal gold antibodies (secondary antibodies, 1:50, v/v; 10-nm gold particles; Sigma-Aldrich) and incubated for 1 h at 37 °C. It was then washed three times with PBST for 5 min each, and twice with d_2_H_2_O for 5 min each. The grid was then stained with uranyl acetate and lead citrate. Control samples were prepared in a similar manner. In control A, pre-immunization serum was used to replace the primary antibody, and in control B the primary antibody was replaced with PBS. The sections were examined and photographed using a Philips Fei-Tecnai 12 TEM.

## Results

### PCD is detected during the formation of secretory cavities

**Fig. 1.**
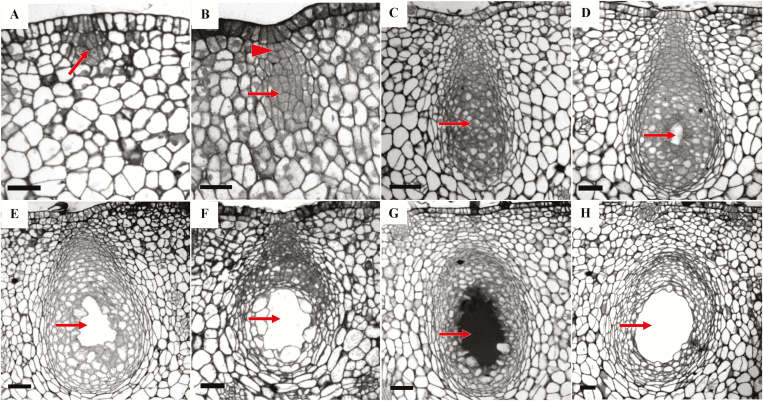
The different developmental stages of the secretory cavity in the fruit of *Citrus grandis* ‘Tomentosa’. (A–C). The initial cell stage. (A) The early stage of the initial cells shows a group of original cells (arrow). (B) The middle stage shows the cap region (arrowhead) and the globular region of the cells (arrow). (C) At the late stage, the cytoplasm in the globular region is no longer dense (arrow). (D) The lumen formation stage of the secretory cavity. The arrow indicates the formation of the secretory space. (E–G). The lumen expansion stage of the secretory cavity. The arrows indicate the lumen. (H). The mature secretory cavity, with the mature lumen indicated by the arrow. The scale bars are 50 μm.

**Fig. 2.**
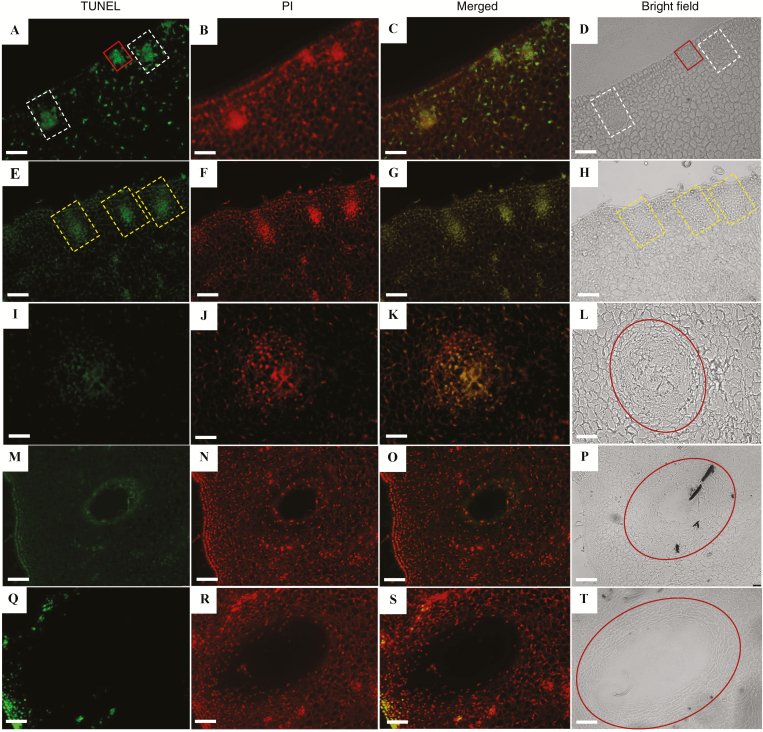
TUNEL assays and propidium iodide (PI) staining of the different developmental stages of the secretory cavity in the fruit of *Citrus grandis* ‘Tomentosa’. (A, E, I, M, Q) TUNEL fluorescence images, where the signal indicates cells undergoing programmed cell death. (B, F, J, N, R) are PI fluorescence images, where the signal indicates cell death. (C, G, K, O, S) Merged TUNEL and PI fluorescence images. (D, H, L, P, T) Bright-field images corresponding to the TUNEL and PI images. In (A, D) the solid boxes indicate the cavities at the early stage of the initial cells and the dashed boxes indicate the cavities at the middle stage. In (E, H) the dashed boxes indicate the cavities at the late stage of initial cells. The circles indicate the cavity at the lumen formation stage (L), at the lumen expansion stage (P), and the mature cavity (T). The scale bars are 100 μm.

The secretory cavities in the fruit of *C.* ‘Tomentosa’ originated from the epidermal and sub-epidermal cells of the ovary wall or of the young fruit. Through division, these formed a group of daughter cells and developed into the early stage of the secretory cavity initial cells (Fig. 1A). These initial cells had denser cytoplasm than the surrounding cells, with a visible TUNEL signal (indicating fragmentation of DNA) and PI fluorescence (indicating dead cells) in the nucleus (Fig. 2A, B). The initial cell groups further differentiated into two distinct parts, one globular (main gland) and one conical (Fig. 1B), and this signified the transition to the middle stage. In this stage, the cells in the center of the globular part were small and polygonal in shape, and the TUNEL signals rapidly increased and became more apparent (Fig. 2A). With the development of the secretory cavity, the staining in the cytoplasm of some cells in the center of the globular part gradually became lighter due to vacuolation, indicating that the initial cells were entering the later stages of development (Fig. 1C). The TUNEL fluorescence signals of several cells in the center of the globular part became slightly weaker compared to the signals from the middle stages, but they were still apparent (Fig. 2E), and PI signals were visible (Fig. 2F). With the development of the cavities, a small gap occurred between 3–4 cells in the center of the globular part, representing the formation stage of the intercellular lumen (Fig. 1D). The TUNEL fluorescence signals became very weak compared to the previous stage (Fig. 2I), and PI signals almost disappeared in the epithelial cells surrounding the lumen of the globular region (Fig. 2J). The secretory lumen then rapidly expanded in diameter, accompanied by the rupture of the inner epithelial cells; this period was hence termed the lumen expansion stage (Fig. 1E–G), at the end of which the mature secretory cavity had developed (Fig. 1H). TUNEL fluorescence signals were not apparent during these two stages (Fig. 2M, Q) and PI fluorescence signals almost disappeared in the degrading epithelial cells (Fig. 2N). PI fluorescence signals became visible again in the epithelial cells of the mature secretory cavity (Fig. 2R).

### *CgCaN* expression and nuclease activity show dynamic changes and tissue specificity at different developmental stages of the secretory cavity

**Fig. 3.**
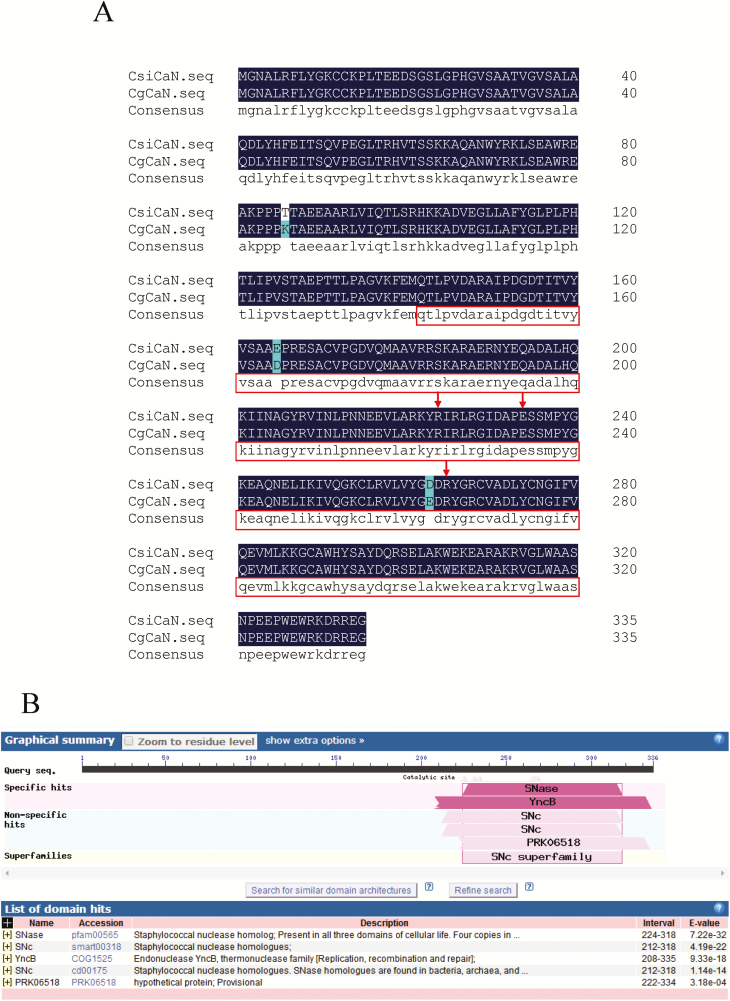
Sequence analyses of *Citrus grandis* CgCaN. (A) Alignment of the deduced amino acid sequences from *Citrus grandis* (CgCaN) and *C. sinensis* (CsiCaN). The boxes indicate thermonuclease domains and the arrows indicate active residues (R-226, E-234, and R-268) as predicted by the Scanprosite software. (B). Functional domain prediction of CgCaN as determined by NCBI Protein BLAST.

A Ca^2+^-dependent DNase gene was cloned from the fruit of *C. grandis* ‘Tomentosa’. The full-length cDNA sequence contained a 1011-bp ORF encoding the 336 aa protein CgCaN. The nucleic acid and amino acid sequences of CgCaN showed ~99% similarities to those of *C. sinensis* (Fig. 3A).

**Fig. 4.**
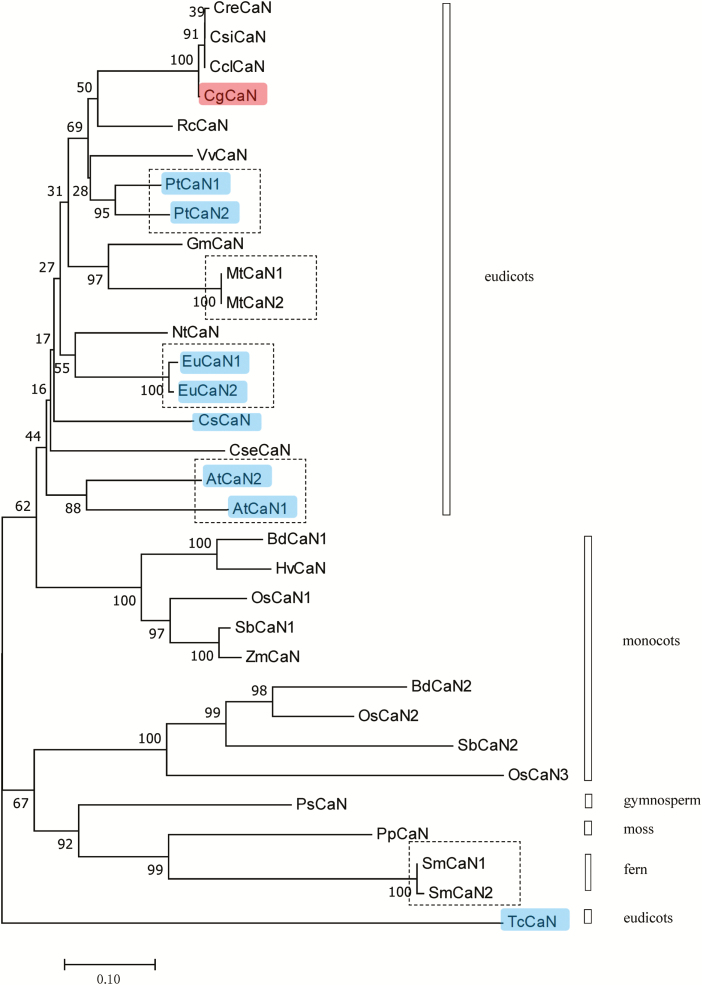
Phylogenetic tree of *Citrus grandis* CgCaN and its homologs obtained from the NCBI database. The tree was analysed using the neighbor-joining method implemented in MEGA version 7.0. Numbers at the branching points indicate the bootstrap proportions (*n*=1000). CgCaN is highlighted, together with the nucleases whose functions have been identified. The dashed boxes indicate nucleases from the same species. At, *Arabidopsis thaliana* (AtCaN1, NP_567 036; AtCaN2, NP_973 649); Bd, *Brachypodium distachyon* (BdCaN1, XP_00 3569 116; BdCaN2, XP_003 577 905); Cg, *Citrus grandis* ‘Tomentosa’; Cre, *Citrus reticulata* Blanco; Cs, *Cucumis sativus* (CsCaN, ABX56707); Cse, *Corydalis sempervirens* (CseCaN, Q39635); Csi, *Citrus sinensis*; Ccl, *Citrus clementina* (CclCaN, XP_006 435 842); Eu, *Eucommia ulmoidies* (EuCaN1, ABA41005; EuCaN2, ACD40019); Gm, *Glycine max* (GmCaN, NP_001 242 780); Hv, *Hordeum vulgare* (HvCaN, BAJ91295); Mt, *Medicago truncatula* (MtCaN1, XP_003 616 736; MtCaN2, XP_003 616 735); Nt, *Nicotiana tabacum* (NtCaN, AAK52082); Os, *Oryza sativa* (OsCaN1, NP_001 042 112; OsCaN2, ABA91143; OsCaN3 = ABA96961); Pp, *Physcomitrella patens* (PpCaN, XP_001 757 699); Ps, *Picea sitchensis* (PsCaN, ABK23214); Pt, *Populus trichocarpa* (PtCaN1, XP_002 311 254; PtCaN2, XP_002 316 184); Rc, *Ricinus communis* (RcCaN, XP_002 520 814); Sb, *Sorghum bicolor* (SbCaN1, XP_002 457 283; SbCaN2, XP_002 441 695); Sm, *Selaginella moellendorffii* (SmCaN1, XP_002 961 000; SmCaN2, XP_002 967 008); Tc, *Theobroma cacao* (TcCaN, XP_007 033 776); Vv, *Vitis vinifera* (VvCan, XP_002 278 465); Zm, *Zea mays* (ZmCaN, ACF87995).

The proposed amino acid sequence for CgCaN was used to search the NCBI database for homologous sequences, and most of them were found to contain a similar SNc conserved region (Tucker *et al.*, 1978). AtCaN in *Arabidopsis thaliana* (Isono *et al.*, 2000), CsCaN in *Cucumis sativus* (Gu *et al.*, 2011), and EuCaN in *Eucommia ulmoides* (Chen *et al.*, 2012) have been reported to be Ca^2+^-dependent DNases. The homologous similarity of CgCAN was 61.01% with AtCaN1, 70.83% with AtCaN2, 66.27% with CsCaN, 70.24% with EuCaN1, 70.54% with EuCaN2, 80.36% with PtCaN1, and 79.17% with PtCaN2 (Supplementary Fig. S2B–H). CgCaN homologs were found in mosses, ferns, gymnosperms, monocots, and eudicots (Fig. 4). Phylogenetic analysis showed that the members in monocots were split into two groups, one of which grouped with eudicots, suggesting a common ancestor. Within the eudicots, there were clusters that suggested lineage-specific gene duplication has occurred. We found that CgCaN had a close phylogenetic relationship with *Ricinus communis* RcCaN, as they were grouped in the same clade.

The calculated molecular weight of CgCaN was 37.57 kDa with a pI of 8.83. Sequence analysis showed that although it had no putative signal peptide, its potential subcellular localization was in the nucleus. Functional domain prediction showed that CgCaN contained a staphylococcal nuclease (SNase-like) domain (Tucker *et al.* 1978) (Fig. 3B). In addition, CgCaN contained the active sites R-226, E-234, and R-268 in the thermonuclease domain (Fig. 3A).

**Fig. 5.**
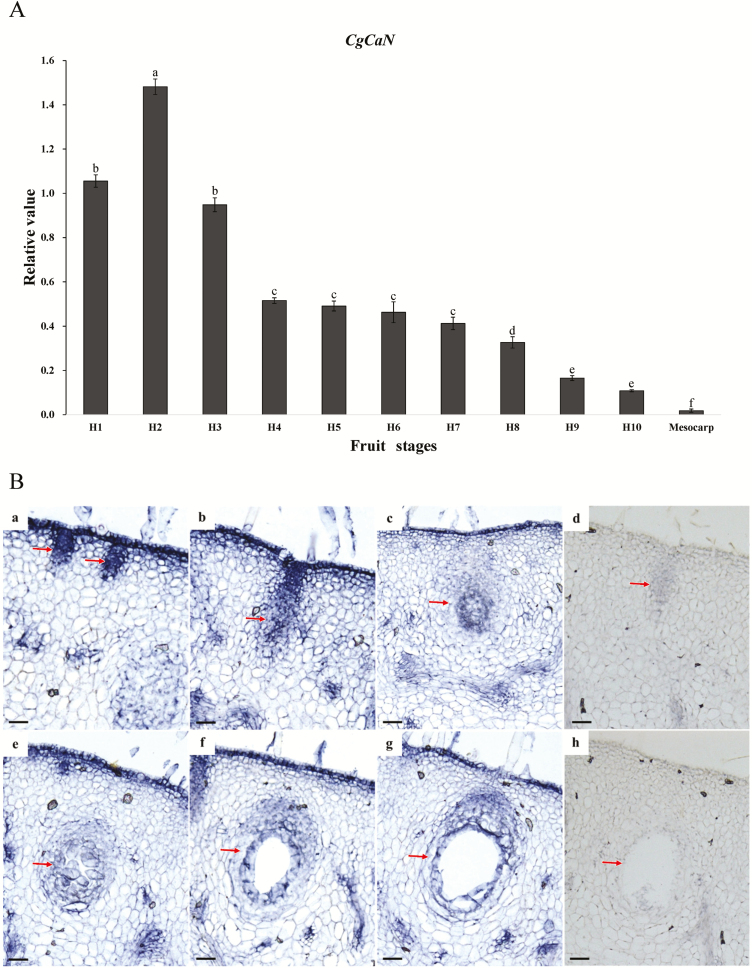
Expression of *Citrus grandis CgCaN* and *in situ* hybridization analysis during fruit development. (A) Expression of *CgCaN* in fruits at progressive developmental stages (see Supplementary Fig. S1) and in mature mesocarp lacking secretory cavities. Values are relative to the expression of *actin*. Different letters indicate significant differences as determined using one-way ANOVA followed by Tukey’s test (*P*<0.05). (B) *In situ* hybridization analysis of the development of secretory cavities in the fruit. (a–c, e–g) Signals during different the different stages of development. Signals were strong in the cells during the early (a) and middle (b) stages of the initial cells, slightly weaker at the late stage (c), and very weak in the secretory cavities at the later developmental stages of the formation (e) and expansion (f) of the lumen, and in the mature cavity (g). (d, h) Negative control. Scale bars are 100 μm.

To investigate the expression patterns of *CgCaN* at different developmental stages of the secretory cavity, we examined the dynamics of transcripts at 10 stages of fruiting using qRT-PCR. The results showed that *CgCaN* expression was high during the early stages, especially during the middle stages of the initial cells (Fig. 5A). The expression level then decreased until the mature stages of the secretory cavity. *CgCaN* expression was very low in mature mesocarp tissue without secretary cavities.

We used *in situ* hybridization to examine potential tissue-specific expression of *CgCaN*, and found that signals mainly occurred in initial cells at the early developmental stages. At the same time, signals were also detected in the vascular bundles and in epidermal cells (Fig. 5B). We focused on the signals in the secretory cavities and found that they were strongest at the early and middle stages of the initial cells (Fig. 5B,a, b) with slightly weaker signals being observed at the late stage (Fig. 5B,c). The signals became very weak during the lumen formation (Fig. 5B,e) and expansion stages (Fig. 5B,f), and completely disappeared at the mature stage (Fig. 5B,g).

**Fig. 6.**
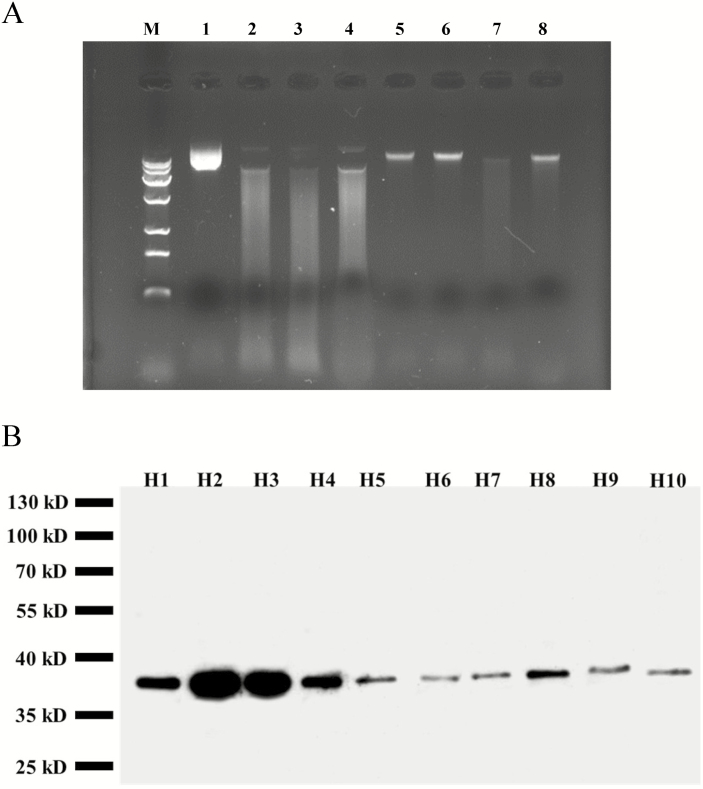
DNase activity of *Citrus grandis* CgCaN fusion proteins expressed in *E. coli*, and western blot analysis of CgCaN at different stages of fruit development. (A) Digestion of genomic DNA by purified His-CgCaN. Lane M, DNA markers. Lanes 1–4, enzymatic detection of plasmid DNA *in vitro*; lanes 5–8, enzymatic detection of genomic DNA from rice *in vitro*. In each case the lanes show a series with the reaction buffer containing 0, 0.001, 0.01, 0.1 M Ca^2+^. (B) Expression of CgCaN in fruits at progressive developmental stages (see Supplementary Fig. S1).

To examine the DNase activity and ionic requirements of CgCaN, the fusion protein with His-tag was expressed in *E. coli*. After 1 h of induction, the His-CgCaN protein appeared in the total proteins. The product increased at 6 h and was purified and mixed with CaCl_2_ for digestion at 37 °C, and then separated on an agarose gel with T65 DNA. The results showed that the His-CgCaN fusion protein could digest plasmid DNA in reaction liquid containing 0.001, 0.01, and 0.1 M calcium ions, and rice genomic DNA in reaction liquid containing 0.01 M calcium ions (Fig. 6A).

Western blotting also showed that CgCaN was expressed at all the developmental stages of the fruit secretory cavity, but in particular during the middle and late stages of the initial cells (Fig. 6B). There was almost no CgCaN expression in mature mesocarp tissue without secretary cavities (Supplementary Fig. S5C).

To determine the source of the calcium ions, we also examined the expression patterns of the CaM gene *PICBP* and the CDPK genes *CDPK5*, *CDPK5-like*, and *CDPK7* (Supplementary Fig. S6). The expression of all these genes initially increased and then decreased with fruit development, showing a similar pattern to the expression of *CgCaN*.

**Fig. 7.**
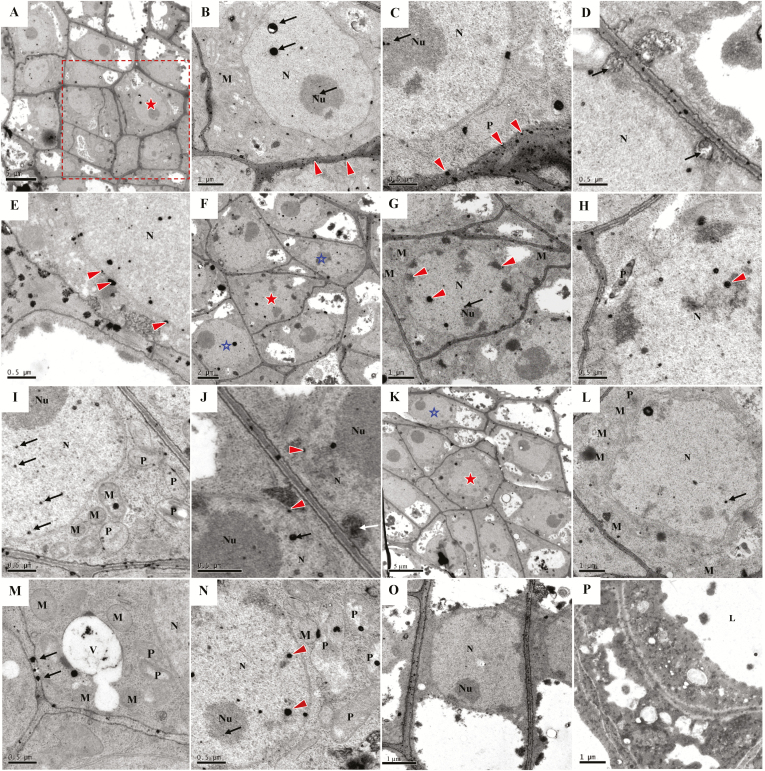
Dynamic variations in Ca^2+^ at the initial cell stage of secretory cavity development in the fruit of *Citrus grandis* ‘Tomentosa’. (A–E) Early stages of the initial cells. (A) The dashed box indicates the globular region. (B–E) Magnified image of the region indicated by the star in (A). (B, C) Ca^2+^ is mainly located in the cell wall (arrowheads), although a few precipitates are present in the cytoplasm, nucleus, plastid, and mitochondria (arrows). Scale bars are (A) 5 μm, (B) 1 μm, and (C) 0.5 μm. (D) Ca^2+^ from the cell wall enters the cytoplasm through pinocytosis (arrows). (E) A number of Ca^2+^ ions (arrowheads) enter the perinuclear space through membrane transport, or through pinocytotic vesicles fused with Ca^2+^ ions that directly fuse with the nuclear membranes. The scale bar is 0.5 μm. (F–J) Middle stage of the initial cells. (F) Cells of the globular region. The peripheral cells develop later than the central cell. (G, H) Magnified images of the region indicated by the solid star in (F). Excess Ca^2+^ accumulates in the nucleus and nucleolus (arrows), specifically in the condensed chromatin (arrowheads). Ca^2+^ ions also appear in the cytoplasm, plastids, and mitochondria. The scale bars are (F) 2 μm, (G) 1 μm, and (H) 0.5 μm. (I, J) Magnified images of the cells indicated by the open stars in (F). The scale bars are 0.5 μm. (I) Ca^2+^ is located in the nucleus (arrow), although a few precipitates are present in the cytoplasm, plastid, and mitochondria. (J) Ca^2+^ ions enter the perinuclear space (arrowheads) and are associated with membrane transport (white arrow). The black arrow indicates Ca^2+^ in the nucleus. (K–N) Late stages of the initial cells. (K) Cells of the globular region. The peripheral cells develop later than the central cells. (L, M) The nucleoli disappear, the nuclear membranes lose their integrity, and Ca^2+^ precipitates rapidly decrease in the residual nuclei (L, arrow) and the cell walls (M, arrows). Ca^2+^ disappears in the cytoplasm, vacuoles, plastids, and mitochondria. The scale bars are (K) 5 μm, (L) 1 μm and (M) 0.5 μm. (N) Magnified images of the cell indicated by the open star in (K). Ca^2+^ accumulates in the nucleus and nucleolus (arrow), specifically in the condensed chromatin (arrowheads). Ca^2+^ ions also appear in the cytoplasm, plastids, and mitochondria. The developmental stage of this cell was the same as in the middle stage, later than the central cell of the globular region. The scale bar is 0.5 μm. (O) The lumen formation stage, with no Ca^2+^ present in the epithelial cells. The scale bar is 1 μm. (P) The lumen expansion stage, with no Ca^2+^ in the epithelial cells. The scale bar is 1 μm. N, nucleus; Nu, nucleolus; V, vacuole; P, plastid; M, mitochondria.

**Fig. 8.**
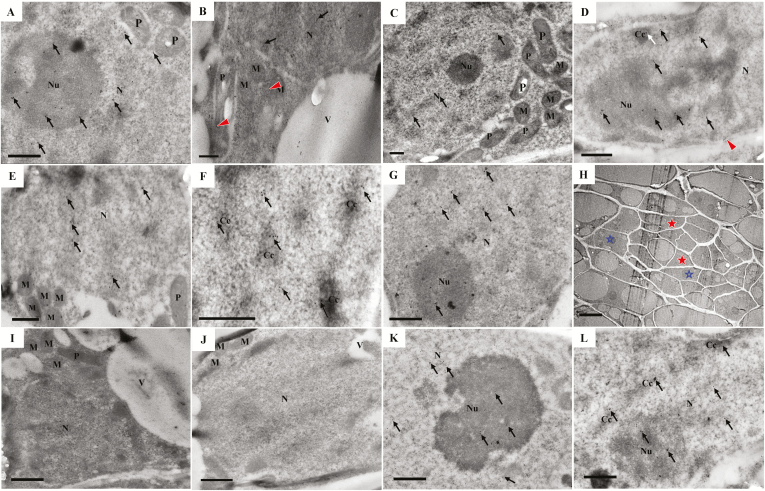
Immunogold localization of CgCaN at the initial cell stage of secretory cavity formation in the fruit of *Citrus grandis* ‘Tomentosa’. (A–C) The early stage of the initial cells. (A, B) In the central cell of the globular region, several gold particles are present in the nuclei and nucleoli (arrows), mitochondria, plastids, and vesicles around the nuclei (B, arrowheads). (C) One of the peripheral cells in the globular region, in which a few gold particles are present in the nuclei and nucleoli (arrows). There are no gold particles in the mitochondria and plastids around the nuclei. The scale bars are 500 nm. (D–G) The middle stage of the initial cells. (D–F) The central cell of the globular region. (D, E) Gold particles are distributed in the nuclei, nucleoli (black arrows), condensed chromatin (white arrow), perinuclear spaces (arrowhead), mitochondria, and plastids. (F) Gold particles are primarily observed in the nucleus, especially in the condensed nuclear chromatin (arrows). (G) One of the peripheral cells in the globular region, with several gold particles present in the nuclei and nucleoli (arrows). The scale bars are 500 nm (H–L) The late stage of the secretory cavity initial cells. The scale bars are 500 nm. (H) The globular region. (I, J) Magnified images of the cells indicated by the solid stars in (H). The nucleoli of the cells in the globular region disappear, nuclear membranes lose their integrity, gold particles in the nuclei rapidly decrease and disappeared, and mitochondria and plastids exhibit no gold particles. (K, L) Magnified images of the cells indicated by the open stars in (H). (K) Several gold particles are present in the nuclei and nucleoli (arrows). (L) The gold particles are primarily observed in the nucleus, especially in condensed nuclear chromatin (arrows). N, nucleus; Nu, nucleolus; Cc, chromatin condensation; V, vacuole; P, plastid; M, mitochondria.

To follow the dynamic changes in calcium ions and CgCaN in the initial cells during development, we used cytochemical and immunocytochemical localization methods, respectively. The TEM results revealed that the spatio-temporal dynamic changes in calcium ion precipitation in the cells of the globular region (Fig. 7B, G, L) during the development of the secretory cavity were consistent with those of CgCaN (Fig. 8A, F, J). The differentiation of the initial cells during apoptosis started from the inner cells and progressed outwards to the peripheral cells. At the early stage of the initial cells, calcium ions in the central cell of the globular region were mainly distributed in the cell wall (Fig 7A), but a small amount of precipitation appeared in the cytoplasm and in the nucleus (Fig. 7B, C). At the same time, large particles of calcium ions were observed to be transported directly across the membrane into the perinuclear space and nucleus (Fig. 7D); moreover, calcium ion precipitation could also be observed in the perinuclear space (Fig. 7E). Calcium ion precipitation also began to occur in the mitochondria and in the plastids (Fig. 7B, C). Correspondingly, the anti-CgCaN immunogold particles in the nucleus and nucleoli of the central cell of the globular region became visible (Fig. 8A), and gold particles were observed in the plastids and mitochondria (Fig. 8B). A few gold particles were observed in the nuclei and nucleoli of the peripheral cells in the globular region (Fig. 8C).

At the middle stage of the secretory cavity initial cells, the most significant morphological characteristic was the large quantity of condensed chromatin in the nuclei of cells in the center of the globular region, which gradually transferred to the edge of the nuclear membrane; these peripheral cells developed slightly later than the central cells (Fig. 7F). At the same time, in the central cells of the globular region, a large number of calcium ions precipitated in the nucleus, especially in the condensed chromatin and nucleoli (Fig. 7F–H). The number of mitochondria and plastids with precipitation calcium also increased (Fig. 7G, H). In contrast, in the peripheral cells of the globular region at the middle stage the cytological characteristics were similar to those of the central cells at the early stage (Fig. 7I, J). During this middle stage, numerous immunogold particles were found to precipitate in the nucleus, especially in the condensed chromatin and nucleoli (Fig. 8D–F), while particles remained in the mitochondria and plastids in the central cell (Fig. 8E). Correspondingly, only a few gold particles were present in the nuclei and nucleoli in the peripheral cells (Fig. 8G).

As the development of the initial cells entered the late stage, condensed chromatin and nucleoli disappeared in the central cell of the globular region (Fig. 7K), the nuclear membrane lost its structure completely, and only the morphology of the residual nucleus remained (Figs 7L, 8I, J). Calcium ion precipitation was then reduced and only a few precipitates were distributed in the residual nucleus (Fig. 7L). In addition, calcium ion precipitation in mitochondria and plastids completely disappeared (Fig. 7M). The anti-CgCaN immunogold particles decreased rapidly and were not readily observed, and the gold particles in the mitochondria and plastids also disappeared (Fig. 8I, J). In the peripheral cells of the globular region, the cytological characteristics were similar to those of the central cell at the middle stage. Calcium ions were observed in the nucleus and nucleolus, specifically in the condensed chromatin (Fig. 7N), whereas the immunogold particles were primarily observed in the nucleus, especially in the condensed nuclear chromatin (Fig. 8K, L). No anti-CgCaN immunogold particles were observed in the secretory cavity of control cells without the CgCaN antibody or in the non-secretory cavity cells of the pericarp (Supplementary Fig. S7).

To determine whether calcium precipitates specifically occurred in PCD cells, we also examined the calcium ion distribution in the non-secretory cavity cells of fruits. We found that normal parenchyma cells of the exocarp had a small amount of calcium precipitation in the cell walls, but no precipitation occurred in the cytoplasm and nuclei of young and mature cells (Fig. S7I, J). This indicated that calcium ions were specifically distributed in the nuclei of PCD cells of the secretory cavity. In addition, we treated samples with the calcium ion chelating agent EGTA and found that most of the precipitates were chelated and that the original precipitation sites became blank (Fig. S7K, L), indicating that the black precipitates observed under TEM were indeed calcium ion precipitation.

## Discussion

### CgCaN is a Ca^2+^-dependent DNase

Zn^2+^- and Ca^2+^-dependent endonucleases are two types of bivalent cationic endonucleases (Ito and Fukuda, 2002) that participate in DNA degradation (Sugiyama *et al.*, 2000). The Zn^2+^-dependent endonuclease ZEN1 can degrade genomic DNA at pH 5.5 during the differentiation of tracheary elements (Ito and Fukuda, 2002). Ca^2+^-dependent nucleases have been found to be involved in the DNA degradation of anther primordia in *Cucumis sativus* and in the differentiation of secondary xylem in *Eucommia ulmoides* (Gu *et al.*, 2011; Chen *et al.*, 2012). Through analysis of DNase activity, we found that a *CgCaN* gene encoding the CgCaN protein with a SNase-like domain was able to degrade rice genomic DNA and plasmid DNA at specific concentrations of calcium ions *in vitro* (Fig. 6A).

At the early stage of the initial cells during PCD of the secretory cavity, TUNEL assays indicated the onset of DNA rupture (Fig. 2), potassium pyroantimonate showed calcium ion precipitation (Fig. 7), and immunogold particle labeling of CgCaN was found to occur in the nucleus (Fig. 8). At the middle stage of the initial cells, abnormal accumulation of calcium ion precipitation in the nucleus was closely associated with the abundance of anti-CgCaN immunogold particles. We therefore conclude that CgCaN is a Ca^2+^-dependent DNase. Although the SNc domains of nuclease genes in eukaryotes are very similar in terms of sequences, the gene with SNc domains have evolved different functions to meet the needs of different biological development processes. For example, Tudor staphylococcal nucleases (Tudor-SN) have both DNase and RNase activities, particularly in the presence of dsRNA during degradation. When RNAi occurs, the nuclease is near the RNA-induced silencing complex (RISC) and performs the function of degrading RNA (Caudy *et al.*, 2003). Two SNc domain nucleases, CaN1 and CaN2, which are Ca^2+^-dependent DNases, have been identified in Arabidopsis (Leśniewicz *et al.*, 2012; Sui *et al.*, 2019). They are modified by N-terminal amino acid residues and are located on the cell membrane, and they might have a defensive role during pathogen invasion (Leśniewicz *et al.*, 2012).

The CgCaN described here is a typical Ca^2+^-dependent DNase and has the SNc domain. Determination of the localization of CgCaN by immunocytochemistry showed that the stage with the most abundant immunogold particles was the same as the stage with peak DNA fragmentation (Figs 2A–D, 8D–F). Gold particles were mainly accumulated in the nucleoli and in the condensed chromatin of the nucleus. The distribution and the dynamics of the particle accumulation were consistent with the TUNEL signals and Ca^2+^ distribution (Figs 2A, 7G, H, 8D–F). We therefore conclude that CgCaN functions in the degradation of genomic DNA in the nucleus during PCD of the secretory cavity cells in the fruit of *C. grandis*, and that it is located in the nucleus.

In plants, Ca^2+^ levels increase as the events of PCD progress, for example in differentiation of tracheary elements, in formation of aerenchyma, in differentiation of aleurone layer cells, during leaf ageing, and in hypersensitivity reactions (Torres *et al.*, 2006). However, the origin of these calcium ions remains unclear. Our present study revealed that during the PCD leading to the formation of secretory cavities in *C. grandis*, Ca^2+^ was first accumulated in the cell wall at the early stage of the initial cells and was gradually transferred into the cytoplasm, before finally accumulating in the nucleus where it reached peak levels at the middle stage (Fig. 7). Ca^2+^ transporters are classified into members of the calmodulin (CaM) and calmodulin-like (CML) protein family, the Ca^2+^-dependent protein kinase (CDPK) family, and the calcineurin B-like protein family (Sanders *et al.*, 2002; Harper and Harmon, 2005; Hashinoto and Kudla, 2011). We selected the CaM gene *PICBP* and the CDPK genes *CDPK5*, *CDPK5-like*, and *CDPK7* for expression analysis and found that changes in their activities were consistent with the dynamics of the calcium ions (Supplementary Fig. S6, Fig. 7). Previous studies have suggested that CaM increases immediately before PCD, and hence the use of calmodulin analogs can prevent cells from entering the PCD stage in *Zinnia elegans* (Kobayashi and Fukuda, 1994). Such findings suggest that calcium and calmodulin systems may be key factors in inducing PCD, and they may regulate it by activating caspase-3 proteases or by stimulating the accumulation of reactive oxygen species and NO (Thomas and Franklin-Tong, 2004; Wilkins *et al.*, 2011). Based on our observations, we conclude that calcium ions mainly come from the cell wall during the formation of secretary cavities in *C. grandis* fruit. In addition, CaM and CDPK might be involved in the transport of Ca^2+^ from the extracellular walls through the cytoplasm into the nucleus (not excluding the mitochondria and plastids) to activate CgCaN for DNA degradation during PCD (Figs 7, 8).

### CgCAN is involved in the PCD process in the formation of secretory cavities in *Citrus grandis* ‘Tomentosa’

The formation of the secretory cavity in *Citrus* fruits is of the schizolysigenous type (Liang *et al.*, 2009), in which PCD is involved in the degradation of the cells (Chen and Wu, 2010; Liu *et al.*, 2012). Zheng *et al.* (2014) reported that the spatio-temporal localization of Ca^2+^ is closely related to changes in the morphology of nuclei, and that it is presumably involved in the regulation of nuclear chromatin and nucleolar degradation during PCD in the fruit secretary cavities of *Citrus sinensis*. In the present study, we further determined the existence of a Ca^2+^-dependent nuclease gene *CgCaN* and its corresponding protein CgCaN. The spatio-temporal expression patterns of the gene and protein coincided with the periods of DNA fragmentation during the PCD process of the development of fruit secretory cavities, as determined by TUNEL assays (Fig. 2), chromatin degradation (Figs 7G, 8D), and nuclear rupture (Figs 7L, 8J). We therefore propose that Ca^2+^-dependent DNases play direct roles in nuclear DNA degeneration during the PCD of secretory cavity cells.

Nuclear degradation is essential in PCD (Obara *et al.*, 2001; He and Kermode, 2003) and nuclear localization is clearly significant for a nuclease that digests genomic DNA (Chen *et al.*, 2012). We found that that Ca^2+^ and CgCaN were mainly localized in the nucleus of degrading cells of the secretory cavity (Figs 7, 8). Moreover, Ca^2+^ precipitation and CgCaN expression peaked before the degradation of chromatin and nucleoli (Figs 6B, 7, 8). Increased concentrations of Ca^2+^ directly activate Ca^2+^-dependent endonucleases, which in turn degrade DNA (Aleksandrushkina and Vanyushin, 2009), strengthening the link between Ca^2+^, CgCaN and the fragmentation of nuclear DNA during the PCD process of secretory cavity development. The accumulation dynamics of CgCaN immunogold-labelled particles in the secretary cavity cells corresponded with the existence and disappearance of chromatin and nucleoli (Fig. 8). In addition, we confirmed that the His-CgCaN fusion protein could digest plasmid DNA and rice genomic DNA *in vitro* at specific calcium ion concentrations (Fig. 6A). Therefore, we believe that CgCaN regulated by Ca^2+^ is involved in nuclear DNA degradation in PCD during the formation of secretory cavities in the fruit of *C. grandis* ‘Tomentosa’. These Ca^2+^-dependent nucleases play important roles in degrading DNA during PCD in plant cell differentiation processes (Sugiyama *et al.*, 2000). Taken together, we conclude that Ca^2+^-dependent DNases are probably conserved in plant PCD processes.

**Fig. 9.**
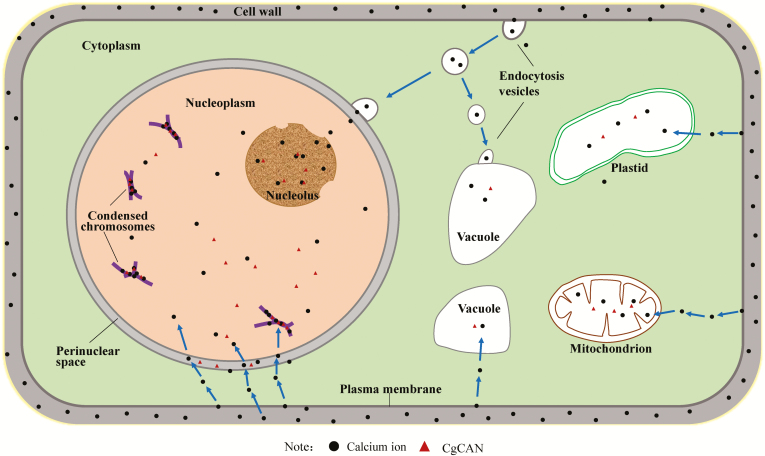
Proposed transport process for calcium ions and CgCaN during fruit development of *Citrus grandis* ‘Tomentosa’. Calcium ions in the cell wall enter the cell by direct diffusion or via endocytosis of the plasma membrane, and are mainly concentrated in the nucleus, nucleolus, condensed chromatin, vacuoles, mitochondria, and plastids. CgCaN is present in the nucleus, nucleolus, condensed chromatin, vacuoles, mitochondria, and plastids.

Mitochondria and plastids are descended from endosymbiont bacteria that have evolved to become essential DNA-containing organelles for cells (Gray, 1993). A calcium/CaM system exists in the chloroplast matrix, (Stael *et al.*, 2012; Chigri *et al.*, 2006), and changes in the concentration of intracellular calcium ions will activate calcium-binding protein kinases on the chloroplast membrane, prompting chloroplasts to participate in intracellular Ca^2+^ regulation (Guo *et al.*, 2016). Previous studies have shown that DPD1, a Mg^2+^-dependent organelle exonuclease, is involved in plastid DNA degradation in Arabidopsis pollen cells (Tang *et al.*, 2012). Endonucleases are also involved in the degradation of mitochondrial DNA in animals (Li *et al.*, 2001; van Loo *et al.*, 2001). In our present study, specific levels of Ca^2+^ and expression of CgCaN were also found to occur in mitochondria and plastids with spatio-temporal coordination. The proposed transport process of Ca^2+^ and role of CgCaN in secretory cavity cells is shown in Fig. 9. We hypothesize that CgCaN could be involved in the DNA fragmentation of mitochondria and plastids, and that this should be investigated in future studies.

## Supplementary data

Supplementary data are available at *JXB* online.

Fig. S1. Definition of the developmental stages H1–H10 in the fruit of *Citrus grandis* ‘Tomentosa’.

Fig. S2. Alignment of the sequence of the candidate Ca^2+^-dependent DNase protein with sequences from different species.

Fig. S3. The amino acid sequence of CgCaN.

Fig. S4. DNase activity of CgCaN fusion proteins expressed in *E. coli* in control samples.

Fig. S5. Verification of antibodies and western blot analysis.

Fig. S6. Expression of *PICBP*, *CDPK5-like*, *CDPK5*, and *CDPK7* at different stages of fruit development.

Fig. S7. Control samples for TUNEL assays and Ca^2+^ precipitation, cells treated with EGTA, immunogold labelling in the absence of antibodies, and immunogold labelling in non-secretory cavity cells.

eraa199_suppl_Supplement_Material

## References

[CIT0001] Aleksandrushkina NI, Vanyushin BF 2009 Endonucleases and their involvement in plant apoptosis. Russian Journal of Plant Physiloogy 56, 291–305.

[CIT0002] Aoyagi S, Sugiyama M, Fukuda H 1998 *BEN1* and *ZEN1* cDNAs encoding S1-type DNases that are associated with programmed cell death in plants. FEBS Letters 429, 134–138. 9650576 10.1016/s0014-5793(98)00563-8

[CIT0003] Asano T, Hayashi N, Kikuchi S, Ohsugi R 2012 CDPK-mediated abiotic stress signaling. Plant Signaling & Behavior 7, 817–821. 22751324 10.4161/psb.20351 PMC3583972

[CIT0004] Batistic O, Kudla J 2009 Plant calcineurin B-like proteins and their interacting protein kinases. Biochimica et Biophysica Acta 1793, 985–992. 19022300 10.1016/j.bbamcr.2008.10.006

[CIT0005] Boudsocq M, Sheen J 2013 CDPKs in immune and stress signaling. Trends in Plant Science 18, 30–40. 22974587 10.1016/j.tplants.2012.08.008 PMC3534830

[CIT0006] Bozhkov PV, Filonova LH, Suarez MF, Helmersson A, Smertenko AP, Zhivotovsky B, von Arnold S 2004 VEIDase is a principal caspase-like activity involved in plant programmed cell death and essential for embryonic pattern formation. Cell Death and Differentiation 11, 175–182. 14576770 10.1038/sj.cdd.4401330

[CIT0007] Caudy AA, Ketting RF, Hammond SM, Denli AM, Bathoorn AM, Tops BB, Silva JM, Myers MM, Hannon GJ, Plasterk RH 2003 A micrococcal nuclease homologue in RNAi effector complexes. Nature 425, 411–414. 14508492 10.1038/nature01956

[CIT0008] Chen HM, Pang Y, Zeng J, Ding Q, Yin SY, Liu C, Lu MZ, Cui KM, He XQ 2012 The Ca^2+^-dependent DNases are involved in secondary xylem development in *Eucommia ulmoides*. Journal of Integrative Plant Biology 54, 456–470. 22694768 10.1111/j.1744-7909.2012.01134.x

[CIT0009] Chen Y, Wu H 2010 Programmed cell death involved in the schizolysigenous formation of the secretory cavity in *Citrus sinensis* L. (Osbeck). Chinese Science Bulletin 55, 2160–2168.

[CIT0010] Chigri F, Hörmann F, Stamp A, Stammers DK, Bölter B, Soll J, Vothknecht UC 2006 Calcium regulation of chloroplast protein translocation is mediated by calmodulin binding to Tic32. Proceedings of the National Academy of Sciences, USA 103, 16051–16056. 10.1073/pnas.0607150103 PMC1635125 17035502

[CIT0011] Coll NS, Epple P, Dangl JL 2011 Programmed cell death in the plant immune system. Cell Death and Differentiation 18, 1247–1256. 21475301 10.1038/cdd.2011.37 PMC3172094

[CIT0012] Dobney S, Chiasson D, Lam P, Smith SP, Snedden WA 2009 The calmodulin-related calcium sensor CML42 plays a role in trichome branching. The Journal of Biological Chemistry 284, 31647–31657. 19720824 10.1074/jbc.M109.056770 PMC2797235

[CIT0013] Domínguez F, Cejudo FJ 2006 Identification of a nuclear-localized nuclease from wheat cells undergoing programmed cell death that is able to trigger DNA fragmentation and apoptotic morphology on nuclei from human cells. The Biochemical Journal 397, 529–536. 16613587 10.1042/BJ20051809 PMC1533310

[CIT0014] Farage-Barhom S, Burd S, Sonego L, Perl-Treves R, Lers A 2008 Expression analysis of the *BFN1* nuclease gene promoter during senescence, abscission, and programmed cell death-related processes. Journal of Experimental Botany 59, 3247–3258. 18603613 10.1093/jxb/ern176 PMC2529240

[CIT0015] Fath A, Bethke P, Lonsdale J, Meza-Romero R, Jones R 2000 Programmed cell death in cereal aleurone. Plant Molecular Biology 44, 255–266. 11199387 10.1023/a:1026584207243

[CIT0016] Fukuda H 2000 Programmed cell death of tracheary elements as a paradigm in plants. Plant Molecular Biology 44, 245–253. 11199386 10.1023/a:1026532223173

[CIT0017] Giacometi S, Marano CA, Bonza MC, Luoni L, Limonta M, De Michelis MI 2012 Phosphorylation of serine residues in the N-terminus modulates the activity of ACA8, a plasma membrane Ca^2+^-ATPase of *Arabidopsis thaliana*. Journal of Experimental Botany 63, 1215–1224. 22090438 10.1093/jxb/err346 PMC3276087

[CIT0018] Gray MW 1993 Origin and evolution of organelle genomes. Current Opinion In Genetics & Development 3, 884–890. 8118213 10.1016/0959-437x(93)90009-e

[CIT0019] Gu HT, Wang DH, Li X, He CX, Xu ZH, Bai SN 2011 Characterization of an ethylene-inducible, calcium-dependent nuclease that is differentially expressed in cucumber flower development. New Phytologist 192, 590–600. 21801181 10.1111/j.1469-8137.2011.03825.x

[CIT0020] Gunawardena AH 2008 Programmed cell death and tissue remodelling in plants. Journal of Experimental Botany 59, 445–451. 17947252 10.1093/jxb/erm189

[CIT0021] Guo H, Feng P, Chi W, et al. 2016 Plastid–nucleus communication involves calcium-modulated MAPK signalling. Nature Communications 7, 12173. 10.1038/ncomms12173 PMC4942575 27399341

[CIT0022] Guo K, Liu S, Takano T, Zhang X 2012 Molecular cloning, expression, and characterization of a Ca^2+^-dependent nuclease of *Arabidopsis thaliana*. Protein Expression and Purification 83, 70–74. 22450164 10.1016/j.pep.2012.03.007

[CIT0023] Hamel LP, Sheen J, Séguin A 2014 Ancient signals: comparative genomics of green plant CDPKs. Trends in Plant Science 19, 79–89. 24342084 10.1016/j.tplants.2013.10.009 PMC3932502

[CIT0024] Hao YJ, Wang DH, Peng YB, Bai SL, Xu LY, Li YQ, Xu ZH, Bai SN 2003 DNA damage in the early primordial anther is closely correlated with stamen arrest in the female flower of cucumber (*Cucumis sativus* L.). Planta 217, 888–895. 12898252 10.1007/s00425-003-1064-x

[CIT0025] Harper JF, Harmon A 2005 Plants, symbiosis and parasites: a calcium signalling connection. Nature Reviews Molecular Cell Biology 6, 555–566. 16072038 10.1038/nrm1679

[CIT0026] Hashimoto K, Kudla J 2011 Calcium decoding mechanisms in plants. Biochimie 93, 2054–2059. 21658427 10.1016/j.biochi.2011.05.019

[CIT0027] He H, Bai M, Tong P, Hu Y, Yang M, Wu H 2018 CELLULASE6 and MANNANASE7 affect cell differentiation and silique dehiscence. Plant Physiology 176, 2186–2201. 29348141 10.1104/pp.17.01494 PMC5841693

[CIT0028] He X, Kermode AR 2003 Nuclease activities and DNA fragmentation during programmed cell death of megagametophyte cells of white spruce (*Picea glauca*) seeds. Plant Molecular Biology 51, 509–521. 12650617 10.1023/a:1022319821591

[CIT0029] Hofius D, Tsitsigiannis DI, Jones JD, Mundy J 2007 Inducible cell death in plant immunity. Seminars in Cancer Biology 17, 166–187. 17218111 10.1016/j.semcancer.2006.12.001

[CIT0030] Isono K, Satoh K, Kobayashi H 2000 Molecular cloning of a cDNA encoding a novel Ca^2+^-dependent nuclease of Arabidopsis that is similar to staphylococcal nuclease. Biochimica et Biophysica Acta - Gene Structure and Expression 1491, 267–272. 10.1016/s0167-4781(00)00007-5 10760589

[CIT0031] Ito J, Fukuda H 2002 ZEN1 is a key enzyme in the degradation of nuclear DNA during programmed cell death of tracheary elements. The Plant Cell 14, 3201–3211. 12468737 10.1105/tpc.006411 PMC151212

[CIT0032] Jiang AL, Cheng Y, Li J, Zhang W 2008 A zinc-dependent nuclear endonuclease is responsible for DNA laddering during salt-induced programmed cell death in root tip cells of rice. Journal of Plant Physiology 165, 1134–1141. 18295371 10.1016/j.jplph.2007.12.008

[CIT0033] Kobayashi H, Fukuda H 1994 Involvement of calmodulin and calmodulin-binding proteins in the differentiation of tracheary elements in *Zinnia* cells. Planta 194, 388–394.

[CIT0034] Landoni M, De Francesco A, Galbiati M, Tonelli C 2010 A loss-of-function mutation in *Calmodulin2* gene affects pollen germination in *Arabidopsis thaliana*. Plant Molecular Biology 74, 235–247. 20683641 10.1007/s11103-010-9669-5

[CIT0035] Leśniewicz K, Poręba E, Smolarkiewicz M, Wolff N, Stanisławski S, Wojtaszek P 2012 Plant plasma membrane-bound staphylococcal-like DNases as a novel class of eukaryotic nucleases. BMC Plant Biology 12, 195. 23102437 10.1186/1471-2229-12-195 PMC3505149

[CIT0036] Li LY, Luo X, Wang X 2001 Endonuclease G is an apoptotic DNase when released from mitochondria. Nature 412, 95–99. 11452314 10.1038/35083620

[CIT0037] Liang SJ, Wang HY, Yang M, Wu H 2009 Sequential actions of pectinases and cellulases during secretory cavity formation in *Citrus* fruits. Trees - Structure and Function 23, 19–27.

[CIT0038] Liang SJ, Wu H, Lun X, Lu D 2006 Secretory cavity development and its relationship with the accumulation of essential oil in fruits of *Citrus medica* L. var. *sarcodactylis* (Noot.) Swingle. Journal of Integrative Plant Biology 48, 573–583.

[CIT0039] Liu PW, Wu H, Yao N, Wu H 2012 Programmed cell death of secretory cavity cells in fruits of *Citrus grandis* cv. Tomentosa is associated with activation of caspase 3-like protease. Trees - Structure and Function 26, 1821–1835.

[CIT0040] Luan S, Lan W, Chul Lee S 2009 Potassium nutrition, sodium toxicity, and calcium signaling: connections through the CBL-CIPK network. Current Opinion in Plant Biology 12, 339–346. 19501014 10.1016/j.pbi.2009.05.003

[CIT0041] McCormack E, Tsai YC, Braam J 2005 Handling calcium signaling: Arabidopsis CaMs and CMLs. Trends in Plant Science 10, 383–389. 16023399 10.1016/j.tplants.2005.07.001

[CIT0042] Mittler R, Shulaev V, Lam E 1995 Coordinated activation of programmed cell death and defense mechanisms in transgenic tobacco plants expressing a bacterial proton pump. The Plant Cell 7, 29–42. 12242350 10.1105/tpc.7.1.29 PMC160762

[CIT0043] Moutinho A, Love J, Trewavas A 1998 Distribution of calmodulin protein and mRNA in growing pollen tubes. Sexual Plant Reproduction 11, 131–139.

[CIT0044] Obara K, Kuriyama H, Fukuda H 2001 Direct evidence of active and rapid nuclear degradation triggered by vacuole rupture during programmed cell death in *Zinnia*. Plant Physiology 125, 615–626. 11161019 10.1104/pp.125.2.615 PMC64863

[CIT0045] Peitsch MC, Mannherz HG, Tschopp J 1994 The apoptosis endonucleases: cleaning up after cell death? Trends in Cell Biology 4, 37–41. 14731864 10.1016/0962-8924(94)90002-7

[CIT0046] Reape TJ, McCabe PF 2010 Apoptotic-like regulation of programmed cell death in plants. Apoptosis 15, 249–256. 20094801 10.1007/s10495-009-0447-2

[CIT0047] Reddy S, Day I S, Narasimhulu SB, Safadi F, Reddy VS, Golovkin M, Harnly MJ 2001 Isolation and characterization of a novel calmodulin-binding protein from potato. The Journal of Biology Chemistry 277, 4206–4214. 10.1074/jbc.M104595200 11684678

[CIT0048] Rogers HJ 2005 Cell death and organ development in plants. Current Topics in Developmental Biology 71, 225–261. 16344107 10.1016/S0070-2153(05)71007-3

[CIT0049] Romeis T, Herde M 2014 From local to global: CDPKs in systemic defense signaling upon microbial and herbivore attack. Current Opinion in Plant Biology 20, 1–10. 24681995 10.1016/j.pbi.2014.03.002

[CIT0050] Sanders D, Pelloux J, Brownlee C, Harper JF 2002 Calcium at the crossroads of signaling. The Plant Cell 14, S401–S417. 12045291 10.1105/tpc.002899 PMC151269

[CIT0051] Simeunovic A, Mair A, Wurzinger B, Teige M 2016 Know where your clients are: subcellular localization and targets of calcium-dependent protein kinases. Journal of Experimental Botany 67, 3855–3872. 27117335 10.1093/jxb/erw157

[CIT0052] Stael S, Wurzinger B, Mair A, Mehlmer N, Vothknecht UC, Teige M 2012 Plant organellar calcium signalling: an emerging field. Journal of Experimental Botany 63, 1525–1542. 22200666 10.1093/jxb/err394 PMC3966264

[CIT0053] Stein JC, Hansen G 1999 Mannose induces an endonuclease responsible for DNA laddering in plant cells. Plant Physiology 121, 71–80. 10482662 10.1104/pp.121.1.71 PMC59391

[CIT0054] Sugiyama M, Ito J, Aoyagi S, Fukuda H 2000 Endonucleases. Plant Molecular Biology 44, 387–397. 11199396 10.1023/a:1026504911786

[CIT0055] Sui W, Guo K, Li L, Liu S, Takano T, Zhang X 2019 Arabidopsis Ca^2+^-dependent nuclease AtCaN2 plays a negative role in plant responses to salt stress. Plant Science 281, 213–222. 30824054 10.1016/j.plantsci.2018.12.007

[CIT0056] Tada Y, Hata S, Takata Y, Nakayashiki H, Tosa Y, Mayama S 2001 Induction and signaling of an apoptotic response typified by DNA laddering in the defense response of oats to infection and elicitors. Molecular Plant-microbe Interactions 14, 477–486. 11310735 10.1094/MPMI.2001.14.4.477

[CIT0057] Tang LY, Matsushima R, Sakamoto W 2012 Mutations defective in ribonucleotide reductase activity interfere with pollen plastid DNA degradation mediated by DPD1 exonuclease. The Plant Journal 70, 637–649. 22239102 10.1111/j.1365-313X.2012.04904.x

[CIT0058] Thomas SG, Franklin-Tong VE 2004 Self-incompatibility triggers programmed cell death in *Papaver* pollen. Nature 429, 305–309. 15152254 10.1038/nature02540

[CIT0059] Tian HQ, Kuang A, Musgrave ME, Russell SD 1998 Calcium distribution in fertile and sterile anthers of a photoperiod sensitive genic male-sterile rice. Planta 204, 183–192.

[CIT0060] Torres MA, Jones JD, Dangl JL 2006 Reactive oxygen species signaling in response to pathogens. Plant Physiology 141, 373–378. 16760490 10.1104/pp.106.079467 PMC1475467

[CIT0061] Tucker PW, Hazen EE Jr, Cotton FA 1978 Staphylococcal nuclease reviewed: a prototypic study in contemporary enzymology. I. Isolation; physical and enzymatic properties. Molecular and Cellular Biochemistry 22, 67–77. 370553 10.1007/BF00496235

[CIT0062] Turner GW, Berrry AM, Gifford EM 1998 Schizogenous secretory cavities of *Citrus limon* (L.) Burm. F. and a re-evaluation of the lysigenous gland concept. International Journal of Plant Sciences 159, 75–88.

[CIT0063] van Loo G, Schotte P, van Gurp M, et al 2001 Endonuclease G: a mitochondrial protein released in apoptosis and involved in caspase-independent DNA degradation. Cell Death and Differentiation 8, 1136–1142. 11753562 10.1038/sj.cdd.4400944

[CIT0064] Wang JJ, Han SF, Li XJ, Gu JT, Lu WJ, Xiao K 2009 Calcium-dependent protein kinases (CPDKs) mediates the molecular basis of plant signal transduction. Acta Prataculturae Sinica 18(3), 241–250.

[CIT0065] Wilkins KA, Bancroft J, Bosch M, Ings J, Smirnoff N, Franklin-Tong VE 2011 Reactive oxygen species and nitric oxide mediate actin reorganization and programmed cell death in the self-incompatibility response of *Papaver*. Plant Physiology 156, 404–416. 21386034 10.1104/pp.110.167510 PMC3091060

[CIT0066] Xu Y, Hanson MR 2000 Programmed cell death during pollination-induced petal senescence in Petunia. Plant Physiology 122, 1323–1333. 10759529 10.1104/pp.122.4.1323 PMC58968

[CIT0067] Young TE, Gallie DR 2000 Programmed cell death during endosperm development. Plant Molecular Biology 44, 283–301. 11199389 10.1023/a:1026588408152

[CIT0068] Zheng P, Bai M, Chen Y, Liu PW, Gao L, Liang SJ, Wu H 2014 Programmed cell death of secretory cavity cells of citrus fruits is associated with Ca^2+^ accumulation in the nucleus. Trees - Structure and Function 28, 1137–1144.

[CIT0069] Zhou L, Fu Y, Yang Z 2009 A genome-wide functional characterization of *Arabidopsis* regulatory calcium sensors in pollen tubes. Journal of Integrative Plant Biology 51, 751–761. 19686372 10.1111/j.1744-7909.2009.00847.x

[CIT0070] Zuppini A, Navazio L, Sella L, Castiglioni C, Favaron F, Mariani P 2005 An endopolygalacturonase from *Sclerotinia sclerotiorum* induces calcium-mediated signaling and programmed cell death in soybean cells. Molecular Plant-Microbe Interactions 18, 849–855. 16134897 10.1094/MPMI-18-0849

