# Supplementary material for: Ca2+-dependent nuclease is involved in DNA degradation during the formation of the secretory cavity by programmed cell death in fruit of Citrus grandis ‘Tomentosa’
Source: J Exp Bot. 2020 Apr 23;71(16):4812–27. doi: 10.1093/jxb/eraa199 (PMC7410178; doi:10.1093/jxb/eraa199)
Supplement: eraa199_suppl_Supplement_Material [file eraa199_suppl_supplement_material.pdf]

A

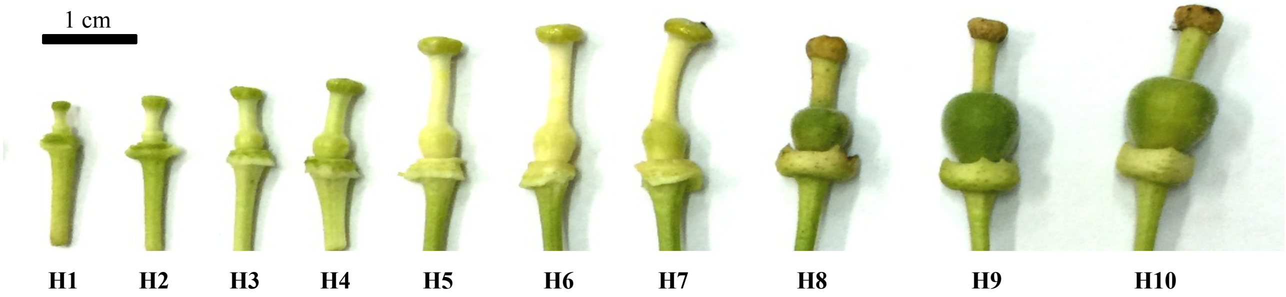

B

Size (mm) (length × width)	2 × 2	3 × 3	4 × 4	6 × 5	7 × 5	8 × 5	8 × 7	10 × 10	13 × 12	17 × 17
stage	H1	H2	H3	H4	H5	H6	H7	H8	H9	H10

C

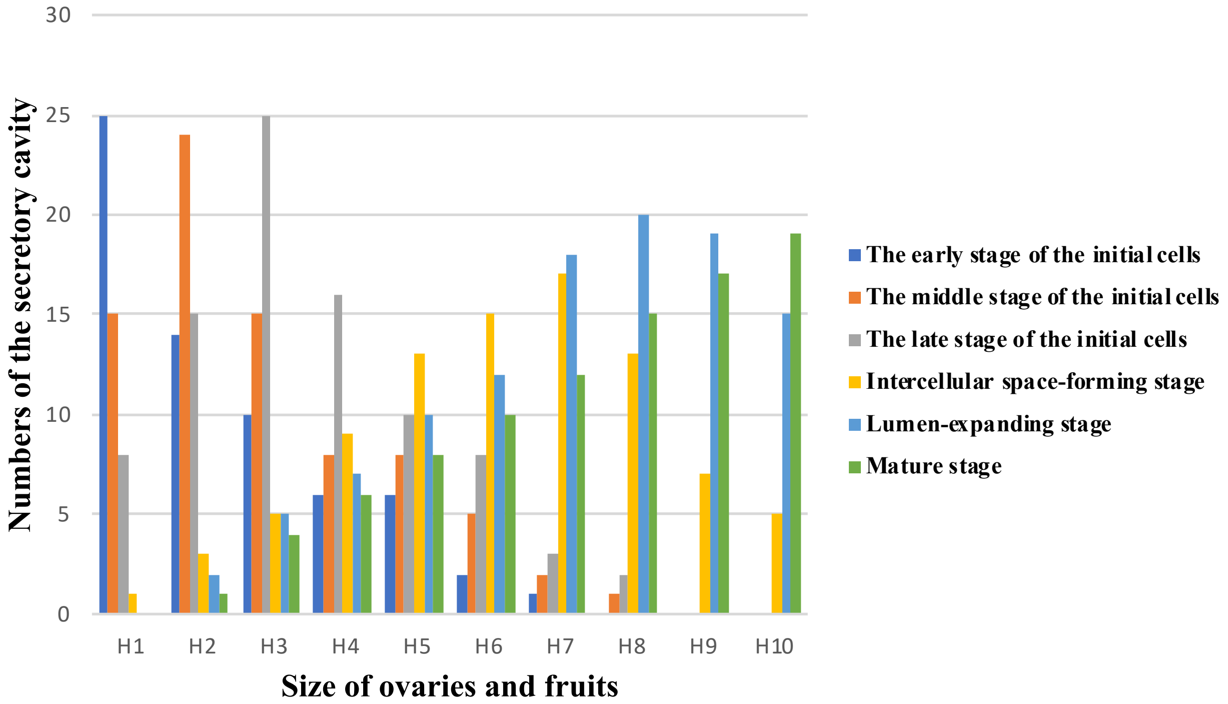

Fig. S1. Statistics for manual sampling standards of secretory cavity development in *Citrus grandis* 'Tomentosa' fruits.

A. Photographs of samples of *Citrus grandis* 'Tomentosa' fruits.

B. The standard for manual sampling of secretory cavity development in *Citrus grandis* 'Tomentosa' fruits.

C. Quantity statistics of secretory cavity developmental stages in different fruit sizes. Sample form: semi-sections (size, 1 mm × 1 mm); Sample quantity: 10.

A

```
AtCaN1.seq   MGNAIRLLRKCLNSHGVSASS...........GGVSALSRDLLNFETTSQVPEKLGSYVVSSQKAQANWYRKIIEAWKQAKPRPKTPEEASRLVIAALK    88
AtCaN2.seq   MGNALTFLYGKCCKPTTTDDS..LGPHGVSAATVGVSALAHDLFNFEITSQVPEGLGRYVQSSRKAQANWYRKIIEAWKQAKPPPQTAEEASRLVTDILK    98
CsCaN.seq    MGNALRFLCGHFSRPTSDSFPP...HAAVSSSTAGVSALPHDLFEFEITSQVPPGLSKHVVSSKKAQANWYAKLAEAWKTEKPTPRTPEEASRLVIQTLK    97
EuCaN1.seq   MGNALRFLYNHCLKPTPEPES.....AGTHV..AGVSALAHDLYHFEITSQVPEGLGGRVVSSKKAQANWYKKISEAWREAKPPPRTPEEVSRLVILTLK    93
EuCaN2.seq   MGNALRFLYNHCFKPTPEQES.....AGTHV..AGVSALAHDLYHFEITSQVPEGLGGHVVSSKKAQANWYKKISEAWREAKPPPRTPEEVSRLVILTLK    93
PtCaN1.seq   MGNALRFLCGHCCKPTEAGDSYSLGPHGVSAATVGVSALARDLLHFEITSQVPEGLGKHVVSSKKAQANWYRKLIEAWKEAKPPPKTPEEAARLVIQTLK   100
PtCaN2.seq   MGNALRFLYGHCCKPTTAGDSDSLGPDGVSAATVGVSALSLDLFHFEITSQVPEGLDKHVVSSKKAQANWFRKLIEAWKEAKPPPETPEEVARLVIQTLK   100
CgCaN.seq    MGNALRFLYGKCCKPLTEEDSGSLGPHGVSAATVGVSALAQDLYHFEITSQVPEGLTRHVTSSKKAQANWYRKLSEAWREAKPPPKTAEEAARLVIQTLS   100
Consensus    mgna   l                        gvsal  dl  fe tsqvp  l    v ss kaqanw  k   eaw   kp p t ee  rlv    l

AtCaN1.seq   NHQKADVEGLLSFYGLPSPHNLVEVP..TEAPVSLPKGVRFELNTLPVDTKSVADGDTVTVYVSSKDPLVSSSLPKDVSLAAVKRAKAREKKNYTEADAL   186
AtCaN2.seq   RNQKADVEGLLSFYGLPLSHTLVEVT..VEAPVSLPEGILFEFQTLPVDPKAVADGDTITVYVSTSEPVVSSSVPREVNLAAVQRAKAREKRNYPKADEL   196
CsCaN.seq    RHQKKDVEGLLTFYGLPLPHTLVKPSAPVPTAVSIPDGVKFELQTLPVDAKAVADGDTVTVYVSTSHPRESSCVPKEVRNAALQRSRARNAKNFAKADAL   197
EuCaN1.seq   RHQKADVEGLLSFYGLPLPHTLVEIS..HGTPPLQPEGLKFELQTLPVDAKAVADGDTITVYVNTIDPRESSSIPREVQMAAVQRSKARSKKNYPKADEL   191
EuCaN2.seq   RHQKADVEGILSFYGLPLPHTLVEIS..HGTPPLQPEGLKFELQTLPVDAKAVADGDTITVYVNTIDPRESSSIPREVQMAAVQRSKARSKKNYPKADEL   191
PtCaN1.seq   RHQKADVEGLLAFYGLPLPHTLIQVS..TGIPASLPEGVKFEMPTLPVDAKAVADGDTITVYVSTTDPRESAYVPREVQTATVQRSKARAERNYGKADAL   198
PtCaN2.seq   RHQKADVEGLLAFYGLQLPHTLAQVS..AGGPTSLPQGVKFEMQTLPVDPKAVADGDTITVYVSTTDTRESSSVPGEVHMAAVQRSKARAERNYGKADAL   198
CgCaN.seq    RHKKADVEGLLAFYGLPLPHTLIPVS..TAEPTTLPAGVKFEMQTLPVDARAIPDGDTITVYVSAADPRESACVPGDVQMAAVRRSKARAERNYEQADAL   198
Consensus      k dveg l fygl   h l              p g  fe  tlpvd      dgdt tvyv        s   p  v   a    r  ar   n   ad l

AtCaN1.seq   HKTIIASGYRMISFQNEEVL.AKKFRIRLSGIDSPESKMPYGKEAHDELLKMVEGKCLKVLVYTEDRYGRCVGDIYCNGKFVQEVMLKKGLAWHYVAYDK   285
AtCaN2.seq   HQKIIDSGYRVLNIENEEVL.ARKFRIRLRGIDAPESQMPFGKEAQEGLLKIVGRKSLKVLVYGEDQYGRCVGDLYCNGIFVQEAMLKKGLAWHYLAYDK   295
CsCaN.seq    HKTITDAGYRVITFQNEEVL.AKKYRIRLRGIDAPESAMPYGKEAKEELKRLVEGKCLRVHVYGEDRYNRCVGDLYCNGKFIQEAMLKKGFAWHYTAYDK   296
EuCaN1.seq   QKKIVDAGYRVLTIQNEDVL.AKKYRIRLRGIDAPESSMPYGKEAKEELTKLVQGKCLRVLVFTEDRYGRCVGDIYCDGIFVQKIMLKKGYAWHYTAYDR   290
EuCaN2.seq   QKKIVDAGYRVLTIQNEDVL.AKKYRIRLRGIDAPESSMPYGKEAKEELTKLVQGKCLRVLVFTEDRYGRCVGDIYCDGIFVQEIMLKKGYAWHYTAYDR   290
PtCaN1.seq   HKKIVEAGYRVLNIQNQEIL.ARKYRIRLRGIDAPESAMPYGKEAKEELANLVQGKCLRIFVYGEDRYGRCVGDIYCNGIFVQEIMLKKGLAWHYTAYDQ   297
PtCaN2.seq   QKKIVEAGYRVINVQNEEIL.ARKYRIRLRGIDAPESSMPYGKEAKEELANLVQGKCLRVFVYGEDRYRRCVGDIYCNGMFVQEVMLKKGLAWHYTAYDK   297
CgCaN.seq    HQKIINAGYRVINLPNNEEVLARKYRIRLRGIDAPESSMPYGKEAQNELIKIVQGKCLRVLVYGEDRYGRCVADLYCNGIFVQEVMLKKGCAWHYSAYDQ   298
Consensus       i   gyr     n      a k rirl gid pes mp gkea    l   v  k l   v   ed y rcv d yc g f q  mlkkg awhy ayd

AtCaN1.seq   RAELAKWENEARQKRVGLWASSNPEKPWEWRKNKRGG   322
AtCaN2.seq   RPVLAKWEKEARQKRIGLWASSNPEKPWDWRKNNRRE   332
CsCaN.seq    RPELSKWENEARAKRAGLWASSNPEQPWEWRKGKREG   333
EuCaN1.seq   RPELEKWEKEARAKRIGLWASSNPEMPWEWRKDRREG   327
EuCaN2.seq   RPELEKWEKEARAKRIGLWASSNPEMPWEWRKDRREG   327
PtCaN1.seq   RWELETWEKEARAKRVGLWASPNPEKPWEWRKDRREG   334
PtCaN2.seq   RRELETWEKEARAKRVGLWASSNPEMPWEWRKDRREG   334
CgCaN.seq    RSELAKWEKEARAKRVGLWAASNPEEPWEWRKDRREG   335
Consensus    r  l   we ear kr glwa  npe pw wrk  r
```

B

```
AtCaN1.seq   ......MGNAIRLLRKCLNSHGVSASSG......GVSALSRDLLNFETTSQVPEKLGSYVVSSQKAQANWYRKIIEAWKQAKPRPKTPEEASRLVIAALK    88
CgCaN.seq    MGNALRFLYGKCCKPLTEEDSGSLGPHGVSAATVGVSALAQDLYHFEITSQVPEGLTRHVTSSKKAQANWYRKLSEAWREAKPPPKTAEEAARLVIQTLS   100
Consensus                         g     g      gvsal  dl  fe tsqvpe l    v ss kaqanwyrk   eaw   akp pkt eea rlvi   l

AtCaN1.seq   NHQKADVEGLLSFYGLPSPHNLVEVPTEAPVSLPKGVRFELNTLPVDTKSVADGDTVTVYVSSKDPLVSSSLPKDVSLAAVKRAKAREKKNYTEADALHK   188
CgCaN.seq    RHKKADVEGLLAFYGLPLPHTLIPVSTAEPTTLPAGVKFEMQTLPVDARAIPDGDTITVYVSAADPRESACVPGDVQMAAVRRSKARAERNYEQADALHQ   200
Consensus     h kadvegll fyglp ph l  v t  p  lp gv fe  tlpvd      dgdt tvyvs  dp  s   p dv  aav r kar   ny  adalh

AtCaN1.seq   TIIASGYRMISFQNEEVL.AKKFRIRLSGIDSPESKMPYGKEAHDELLKMVEGKCLKVLVYTEDRYGRCVGDIYCNGKFVQEVMLKKGLAWHYVAYDKRA   287
CgCaN.seq    KIINAGYRVINLPNNEEVLARKYRIRLRGIDAPESSMPYGKEAQNELIKIVQGKCLRVLVYGEDRYGRCVADLYCNGIFVQEVMLKKGCAWHYSAYDQRS   300
Consensus     ii  gyr i    n e   a k rirl gid pes mpygkea  el k v gkcl vlvy edrygrcv d ycng fvqevmlkkg awhy ayd r

AtCaN1.seq   ELAKWENEARQKRVGLWASSNPEKPWEWRKNKRGG   322
CgCaN.seq    ELAKWEKEARAKRVGLWAASNPEEPWEWRKDRREG   335
Consensus    elakwe ear krvglwa snpe pwewrk  r g
```

C

```
AtCaN2.seq   MGNALTFLYGKCCKPTTTDDS..LGPHGVSAATVGVSALAHDLFNFEITSQVPEGLGRYVQSSRKAQANWYRKIIEAWKQAKPPPQTAEEASRLVTDILK    98
CgCaN.seq    MGNALRFLYGKCCKPLTEEDSGSLGPHGVSAATVGVSALAQDLYHFEITSQVPEGLTRHVTSSKKAQANWYRKLSEAWREAKPPPKTAEEAARLVIQTLS   100
Consensus    mgnal flygkcckp t  ds  lgphgvsaatvgvsala dl  feitsqvpegl r v ss kaqanwyrk   eaw   akppp taeea rlv    l

AtCaN2.seq   RNQKADVEGLLSFYGLPLSHTLVEVTVEAPVSLPEGILFEFQTLPVDPKAVADGDTITVYVSTSEPVVSSSVPREVNLAAVQRAKAREKRNYPKADELHQ   198
CgCaN.seq    RHKKADVEGLLAFYGLPLPHTLIPVSTAEPTTLPAGVKFEMQTLPVDARAIPDGDTITVYVSAADPRESACVPGDVQMAAVRRSKARAERNYEQADALHQ   200
Consensus    r  kadvegll fyglpl htl  v     p  lp g  fe qtlpvd  a  dgdtitvyvs   p  s  vp  v  aav r kar  rny  ad lhq

AtCaN2.seq   KIIDSGYRVLNIENEEVL.ARKFRIRLRGIDAPESQMPFGKEAQEGLLKIVGRKSLKVLVYGEDQYGRCVGDLYCNGIFVQEAMLKKGLAWHYLAYDKRP   297
CgCaN.seq    KIINAGYRVINLPNNEEVLARKYRIRLRGIDAPESSMPYGKEAQNELIKIVQGKCLRVLVYGEDRYGRCVADLYCNGIFVQEVMLKKGCAWHYSAYDQRS   300
Consensus    kii  gyrv n   n e   ark rirlrgidapes mp gkeaq  l kiv  k l vlvyged ygrcv dlycngifvqe mlkkg awhy ayd r

AtCaN2.seq   VLAKWEKEARQKRIGLWASSNPEKPWDWRKNNRRE   332
CgCaN.seq    ELAKWEKEARAKRVGLWAASNPEEPWEWRKDRREG   335
Consensus     lakwekear kr glwa snpe pw wrk  r
```

D

```
CsCaN.seq   MGNALRFLCGHF.SRPTSDSFPPHAAVSSSTA..GVSALPHDLFEFEITSQVPPGLSKHVVSSKKAQANWYAKLAEAWKTEKPTPRTPEEASRLVIQTLK    97
CgCaN.seq   MGNALRFLYGKCCKPLTEEDSGSLGPHGVSAATVGVSALAQDLYHFEITSQVPEGLTRHVTSSKKAQANWYRKLSEAWREAKPPPKTAEEAARLVIQTLS   100
Consensus   mgnalrfl g      t            s a  gvsal  dl  feitsqvp gl  hv sskkaqanwy kl eaw   kp p t eea rlviqtl

CsCaN.seq   RHQKKDVEGLLTFYGLPLPHTLVKPSAPVPTAVSIPDGVKFELQTLPVDAKAVADGDTVTVYVSTSHPRESSCVPKEVRNAALQRSRARNAKNFAKADAL   197
CgCaN.seq   RHKKADVEGLLAFYGLPLPHTLIPVSTAEPTTLPA..GVKFEMQTLPVDARAIPDGDTITVYVSAADPRESACVPGDVQMAAVRRSKARAERNYEQADAL   198
Consensus   rh k dvegll fyglplphtl   s   pt      gvkfe qtlpvda a  dgdt tvyvs   pres cvp  v  aa  rs ar   n   adal

CsCaN.seq   HKTITDAGYRVITFQNEEVL.AKKYRIRLRGIDAPESAMPYGKEAKEELKRLVEGKCLRVHVYGEDRYNRCVGDLYCNGKFIQEAMLKKGFAWHYTAYDK   296
CgCaN.seq   HQKIINAGYRVINLPNNEEVLARKYRIRLRGIDAPESSMPYGKEAQNELIKIVQGKCLRVLVYGEDRYGRCVADLYCNGIFVQEVMLKKGCAWHYSAYDQ   298
Consensus   h  i  agyrvi   n e   a kyrirlrgidapes mpygkea  el   v gkclrv vygedry rcv dlycng f qe mlkkg awhy ayd

CsCaN.seq   RPELSKWENEARAKRAGLWASSNPEQPWEWRKGKREG                                                                 333
CgCaN.seq   RSELAKWEKEARAKRVGLWAASNPEEPWEWRKDRREG                                                                 335
Consensus   r el kwe earakr glwa snpe pwewrk  reg
```

E

```
EuCaN1.seq  MGNALRFLYNHC.LKPTPEPESAGTHVA......GVSALAHDLYHFEITSQVPEGLGGRVVSSKKAQANWYKKISEAWREAKPPPRTPEEVSRLVILTLK    93
CgCaN.seq   MGNALRFLYGKCCKPLTEEDSGSLGPHGVSAATVGVSALAQDLYHFEITSQVPEGLTRHVTSSKKAQANWYRKLSEAWREAKPPPKTAEEAARLVIQTLS   100
Consensus   mgnalrfly  c    t e               gvsala dlyhfeitsqvpegl   v sskkaqanwy k seawreakppp t ee  rlvi tl

EuCaN1.seq  RHQKADVEGLLSFYGLPLPHTLVEISHGTPPLQPEGLKFELQTLPVDAKAVADGDTITVYVNTIDPRESSSIPREVQMAAVQRSKARSKKNYPKADELQK   193
CgCaN.seq   RHKKADVEGLLAFYGLPLPHTLIPVSTAEPTTLPAGVKFEMQTLPVDARAIPDGDTITVYVSAADPRESACVPGDVQMAAVRRSKARAERNYEQADALHQ   200
Consensus   rh kadvegll fyglplphtl   s   p   p g kfe qtlpvda a  dgdtitvyv   dpres   p  vqmaav rskar   ny  ad l

EuCaN1.seq  KIVDAGYRVLTIQNEDVL.AKKYRIRLRGIDAPESSMPYGKEAKEELTKLVQGKCLRVLVFTEDRYGRCVGDIYCDGIFVQKIMLKKGYAWHYTAYDRRP   292
CgCaN.seq   KIINAGYRVINLPNNEEVLARKYRIRLRGIDAPESSMPYGKEAQNELIKIVQGKCLRVLVYGEDRYGRCVADLYCNGIFVQEVMLKKGCAWHYSAYDQRS   300
Consensus   ki  agyrv    n     a kyrirlrgidapessmpygkea  el k vqgkclrvlv  edrygrcv d yc gifvq  mlkkg awhy ayd r

EuCaN1.seq  ELEKWEKEARAKRIGLWASSNPEMPWEWRKDRREG                                                                   327
CgCaN.seq   ELAKWEKEARAKRVGLWAASNPEEPWEWRKDRREG                                                                   335
Consensus   el kwekearakr glwa snpe pwewrkdrreg
```

F

```
EuCaN2.seq  MGNALRFLYNHC.FKPTPEQESAGTHVA......GVSALAHDLYHFEITSQVPEGLGGHVVSSKKAQANWYKKISEAWREAKPPPRTPEEVSRLVILTLK    93
CgCaN.seq   MGNALRFLYGKCCKPLTEEDSGSLGPHGVSAATVGVSALAQDLYHFEITSQVPEGLTRHVTSSKKAQANWYRKLSEAWREAKPPPKTAEEAARLVIQTLS   100
Consensus   mgnalrfly  c    t e               gvsala dlyhfeitsqvpegl  hv sskkaqanwy k seawreakppp t ee  rlvi tl

EuCaN2.seq  RHQKADVEGILSFYGLPLPHTLVEISHGTPPLQPEGLKFELQTLPVDAKAVADGDTITVYVNTIDPRESSSIPREVQMAAVQRSKARSKKNYPKADELQK   193
CgCaN.seq   RHKKADVEGLLAFYGLPLPHTLIPVSTAEPTTLPAGVKFEMQTLPVDARAIPDGDTITVYVSAADPRESACVPGDVQMAAVRRSKARAERNYEQADALHQ   200
Consensus   rh kadveg l fyglplphtl   s   p   p g kfe qtlpvda a  dgdtitvyv   dpres   p  vqmaav rskar   ny  ad l

EuCaN2.seq  KIVDAGYRVLTIQNEDVL.AKKYRIRLRGIDAPESSMPYGKEAKEELTKLVQGKCLRVLVFTEDRYGRCVGDIYCDGIFVQEIMLKKGYAWHYTAYDRRP   292
CgCaN.seq   KIINAGYRVINLPNNEEVLARKYRIRLRGIDAPESSMPYGKEAQNELIKIVQGKCLRVLVYGEDRYGRCVADLYCNGIFVQEVMLKKGCAWHYSAYDQRS   300
Consensus   ki  agyrv    n     a kyrirlrgidapessmpygkea  el k vqgkclrvlv  edrygrcv d yc gifvqe mlkkg awhy ayd r

EuCaN2.seq  ELEKWEKEARAKRIGLWASSNPEMPWEWRKDRREG                                                                   327
CgCaN.seq   ELAKWEKEARAKRVGLWAASNPEEPWEWRKDRREG                                                                   335
Consensus   el kwekearakr glwa snpe pwewrkdrreg
```

G

```
PtCaN1.seq  MGNALRFLCGHCCKPTEAGDSYSLGPHGVSAATVGVSALARDLLHFEITSQVPEGLGKHVVSSKKAQANWYRKLLEAWKEAKPPPKTPEEAARLVIQTLK   100
CgCaN.seq   MGNALRFLYGKCCKPLTEEDSGSLGPHGVSAATVGVSALAQDLYHFEITSQVPEGLTRHVTSSKKAQANWYRKLSEAWREAKPPPKTAEEAARLVIQTLS   100
Consensus   mgnalrfl g cckp    ds slgphgvsaatvgvsala dl hfeitsqvpegl  hv sskkaqanwyrkl eaw eakpppkt eeaarlviqtl

PtCaN1.seq  RHQKADVEGLLAFYGLPLPHTLIQVSTGIPASLPEGVKFEMPTLPVDAKAVADGDTITVYVSTTDPRESAYVPREVQTATVQRSKARAERNYGKADALHK   200
CgCaN.seq   RHKKADVEGLLAFYGLPLPHTLIPVSTAEPTTLPAGVKFEMQTLPVDARAIPDGDTITVYVSAADPRESACVPGDVQMAAVRRSKARAERNYEQADALHQ   200
Consensus   rh kadvegllafyglplphtli vst  p  lp gvkfem tlpvda a  dgdtitvyvs  dpresa vp  vq a v rskaraerny  adalh

PtCaN1.seq  KIVEAGYRVLNIQNQEIL.ARKYRIRLRGIDAPESAMPYGKEAKEELANLVQGKCLRIFVYGEDRYGRCVGDIYCNGIFVQEIMLKKGLAWHYTAYDQRW   299
CgCaN.seq   KIINAGYRVINLPNNEEVLARKYRIRLRGIDAPESSMPYGKEAQNELIKIVQGKCLRVLVYGEDRYGRCVADLYCNGIFVQEVMLKKGCAWHYSAYDQRS   300
Consensus   ki  agyrv n  n e   arkyrirlrgidapes mpygkea  el   vqgkclr  vygedrygrcv d ycngifvqe mlkkg awhy aydqr

PtCaN1.seq  ELETWEKEARAKRVGLWASPNPEKPWEWRKDRREG                                                                   334
CgCaN.seq   ELAKWEKEARAKRVGLWAASNPEEPWEWRKDRREG                                                                   335
Consensus   el  wekearakrvglwa  npe pwewrkdrreg
```

H

```
PtCaN2.seq  MGNALRFLYGHCCKPTTAGDSDSLGPDGVSAATVGVSALSLDLFHFEITSQVPEGLDKHVVSSKKAQANWFRKLLEAWKEAKPPPETPEEVARLVIQTLK   100
CgCaN.seq   MGNALRFLYGKCCKPLTEEDSGSLGPHGVSAATVGVSALAQDLYHFEITSQVPEGLTRHVTSSKKAQANWYRKLSEAWREAKPPPKTAEEAARLVIQTLS   100
Consensus   mgnalrflyg cckp t  ds slgp gvsaatvgvsal  dl hfeitsqvpegl  hv sskkaqanw rkl eaw eakppp t ee arlviqtl

PtCaN2.seq  RHQKADVEGLLAFYGLQLPHTLAQVSAGGPTSLPQGVKFEMQTLPVDPKAVADGDTITVYVSTTDTRESSSVPGEVHMAAVQRSKARAERNYGKADALQK   200
CgCaN.seq   RHKKADVEGLLAFYGLPLPHTLIPVSTAEPTTLPAGVKFEMQTLPVDARAIPDGDTITVYVSAADPRESACVPGDVQMAAVRRSKARAERNYEQADALHQ   200
Consensus   rh kadvegllafygl lphtl  vs   pt lp gvkfemqtlpvd  a  dgdtitvyvs  d res  vpg v maav rskaraerny  adal

PtCaN2.seq  KIVEAGYRVINVQNEEIL.ARKYRIRLRGIDAPESSMPYGKEAKEELANLVQGKCLRVFVYGEDRYRRCVGDIYCNGMFVQEVMLKKGLAWHYTAYDKRR   299
CgCaN.seq   KIINAGYRVINLPNNEEVLARKYRIRLRGIDAPESSMPYGKEAQNELIKIVQGKCLRVLVYGEDRYGRCVADLYCNGIFVQEVMLKKGCAWHYSAYDQRS   300
Consensus   ki  agyrvin  n e   arkyrirlrgidapessmpygkea  el   vqgkclrv vygedry rcv d ycng fvqevmlkkg awhy ayd r

PtCaN2.seq  ELETWEKEARAKRVGLWASSNPEMPWEWRKDRREG                                                                   334
CgCaN.seq   ELAKWEKEARAKRVGLWAASNPEEPWEWRKDRREG                                                                   335
Consensus   el  wekearakrvglwa snpe pwewrkdrreg
```

Fig. S2. Alignment of the different Ca^{2+}-dependent DNase protein.

A. Alignment of the candidate Ca^{2+}-dependent DNase CgCaN and published plant Ca^{2+}-dependent DNases.

B. Alignment of CgCaN and AtCaN1.

C. Alignment of CgCaN and AtCaN2.

D. Alignment of CgCaN and CsCaN.

E. Alignment of CgCaN and EuCaN1.

F. Alignment of CgCaN and EuCaN2.

G. Alignment of CgCaN and ptCaN1.

H. Alignment of CgCaN and PtCaN2.

SciCaN, *Citrus sinensis*; PtCaN, *Populus trichocarpa*; EuCaN, *Eucommia ulmoidies*; AtCaN, *Arabidopsis thaliana*; CsaCaN, *Cucumis sativus*

Translation of CgCaN(1-1011)

```
                10        20        30        40        50        60
1       ATGGGAAATGCACTTAGGTTCCTTTACGGCAAATGTTGCAAGCCCTTAACTGAAGAGGAC
1        M  G  N  A  L  R  F  L  Y  G  K  C  C  K  P  L  T  E  E  D

                70        80        90       100       110       120
61      TCTGGATCACTTGGTCCTCATGGTGTCTCTGCTGCCACCGTTGGTGTCTCAGCTCTTGCT
21       S  G  S  L  G  P  H  G  V  S  A  A  T  V  G  V  S  A  L  A

               130       140       150       160       170       180
121     CAAGATCTCTACCACTTTGAGATCACCTCACAGGTCCCAGAAGGGCTCACTAGGCATGTT
41       Q  D  L  Y  H  F  E  I  T  S  Q  V  P  E  G  L  T  R  H  V

               190       200       210       220       230       240
181     ACATCTTCAAAGAAGGCTCAGGCTAATTGGTATAGAAAACTCTCAGAGGCATGGAGAGAA
61       T  S  S  K  K  A  Q  A  N  W  Y  R  K  L  S  E  A  W  R  E

               250       260       270       280       290       300
241     GCGAAACCGCCTCCAAAAACGGCAGAGGAAGCAGCTAGGCTCGTGATTCAGACCCTATCG
81       A  K  P  P  P  K  T  A  E  E  A  A  R  L  V  I  Q  T  L  S

               310       320       330       340       350       360
301     AGACACAAAAAGGCCGATGTTGAGGGTTTGTTGGCTTTCTATGGCTTGCCTCTTCCTCAT
101      R  H  K  K  A  D  V  E  G  L  L  A  F  Y  G  L  P  L  P  H

               370       380       390       400       410       420
361     ACCCTTATTCCAGTTTCTACTGCAGAACCAACTACACTGCCAGCGGGAGTTAAGTTTGAA
121      T  L  I  P  V  S  T  A  E  P  T  T  L  P  A  G  V  K  F  E

               430       440       450       460       470       480
421     ATGCAGACACTACCGGTTGATGCAAGAGCAATACCAGATGGGGATACCATAACCGTGTAT
141      M  Q  T  L  P  V  D  A  R  A  I  P  D  G  D  T  I  T  V  Y

               490       500       510       520       530       540
481     GTTAGCGCGGCTGATCCAAGGGAGTCAGCATGTGTTCCTGGAGACGTACAAATGGCTGCT
161      V  S  A  A  D  P  R  E  S  A  C  V  P  G  D  V  Q  M  A  A
```


```
               490       500       510       520       530       540
481     GTTAGCGCGGCTGATCCAAGGGAGTCAGCATGTGTTCCTGGAGACGTACAAATGGCTGCT
161      V  S  A  A  D  P  R  E  S  A  C  V  P  G  D  V  Q  M  A  A
```


```
               550       560       570       580       590       600
541     GTTCGAAGATCAAAAGCACGAGCTGAGAGAAACTATGAACAAGCAGATGCACTTCACCAG
181      V  R  R  S  K  A  R  A  E  R  N  Y  E  Q  A  D  A  L  H  Q

               610       620       630       640       650       660
601     AAAATCATCAATGCTGGATACCGGGTGATAAATCTTCCAAACAACGAGGAAGTTCTAGCA
201      K  I  I  N  A  G  Y  R  V  I  N  L  P  N  N  E  E  V  L  A

               670       680       690       700       710       720
661     CGAAAGTATCGAATTCGACTAAGAGGCATAGATGCACCAGAGAGTTCAATGCCATATGGG
221      R  K  Y  R  I  R  L  R  G  I  D  A  P  E  S  S  M  P  Y  G

               730       740       750       760       770       780
721     AAAGAAGCACAGAATGAACTGATTAAGATTGTTCAAGGAAAGTGTTTGAGAGTCCTAGTC
241      K  E  A  Q  N  E  L  I  K  I  V  Q  G  K  C  L  R  V  L  V

               790       800       810       820       830       840
781     TATGGTGAGGATCGTTATGGTCGCTGCGTAGCAGATTTGTATTGCAATGGCATATTTGTA
261      Y  G  E  D  R  Y  G  R  C  V  A  D  L  Y  C  N  G  I  F  V

               850       860       870       880       890       900
841     CAGGAAGTGATGCTCAAGAAAGGGTGTGCATGGCATTACTCTGCCTATGACCAACGCTCA
281      Q  E  V  M  L  K  K  G  C  A  W  H  Y  S  A  Y  D  Q  R  S

               910       920       930       940       950       960
901     GAACTTGCAAAATGGGAAAAAGAGGCTCGAGCAAAGAGAGTTGGGTTATGGGCAGCATCA
301      E  L  A  K  W  E  K  E  A  R  A  K  R  V  G  L  W  A  A  S

               970       980       990      1000      1010
961     AACCCAGAAGAGCCATGGGAATGGAGAAAGGACAGGCGCGAAGGCCGATAG
321      N  P  E  E  P  W  E  W  R  K  D  R  R  E  G  R  *
```

Fig. S3. The amino acid sequence of CgCaN.

The 1011 bp cDNA sequence was translated into its corresponding protein sequence using the DNAMAN8.0 software.

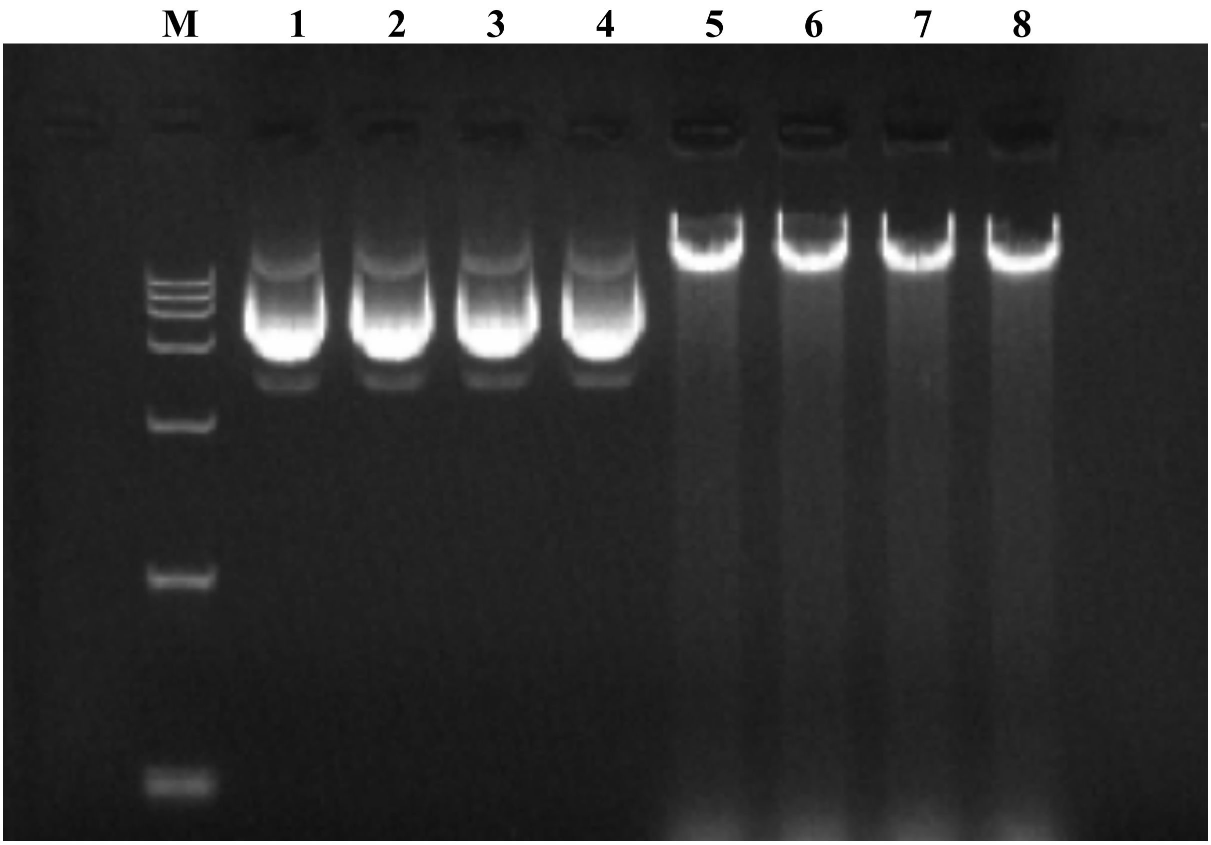
M 1 2 3 4 5 6 7 8

Fig. S4. The controls of DNase activity analysis of CgCaN fusion proteins expressed in *E. coli*, Digestion of genomic DNA without purified His-CgCaN in tube. Lane M, DNA marker; lanes 1 to 4: enzymatic detection of plasmid DNA *in vitro*, reaction buffer contained 0, 0.001, 0.01, 0.1 M Ca^{2+}; lanes 5 to 8: enzymatic detection of genomic DNA from rice *in vitro*, reaction buffer contained 0, 0.001, 0.01, 0.1 M Ca^{2+}.

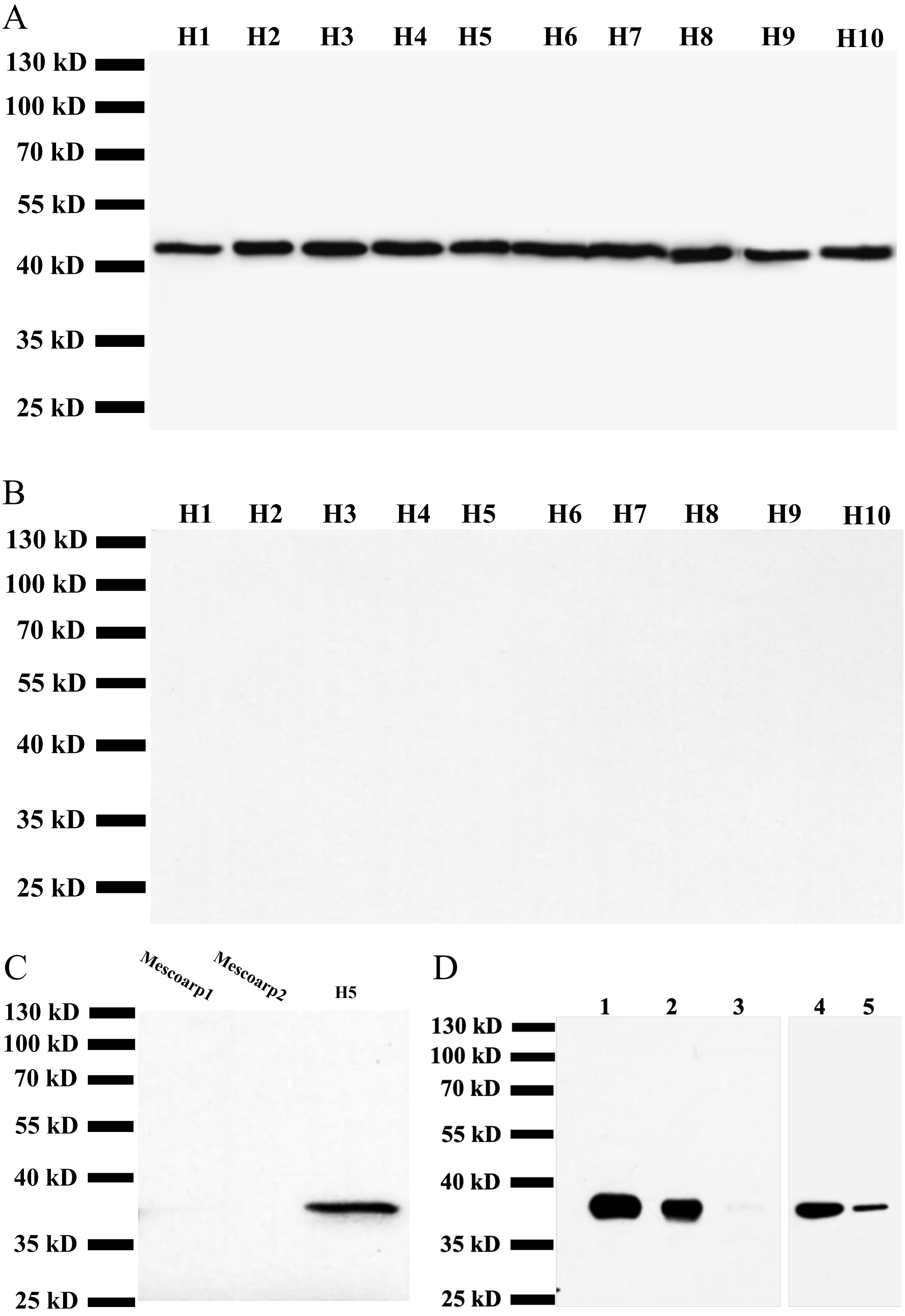

A
H1 H2 H3 H4 H5 H6 H7 H8 H9 H10
130 kD
100 kD
70 kD
55 kD
40 kD
35 kD
25 kD
B
H1 H2 H3 H4 H5 H6 H7 H8 H9 H10
130 kD
100 kD
70 kD
55 kD
40 kD
35 kD
25 kD
C
Mescoarp1
Mescoarp2
H5
130 kD
100 kD
70 kD
55 kD
40 kD
35 kD
25 kD
D
1 2 3 4 5
130 kD
100 kD
70 kD
55 kD
40 kD
35 kD
25 kD

Fig. S5. Antibody verification and western blot analysis.

A. The internal controls *(β-actin)* have the same expression levels in all sizes of fruits.

B. The controls of the western blot analysis. There is no CgCaN expression in any line.

C. The western blot analysis of the non-cavities tissues (mesocarp), there is almost no CgCaN expression in line1 and line2.

D. The exogenous and endogenous proteins are used to detect antibodies. Exogenous proteins were from the expression of recombinant plasmids pET-28a *in vitro*, endogenous proteins were from the fruit exocarps of *C. grandis* 'Tomentosa'. 1, 10 ng expressed protein; 2, 5 ng expressed protein; 3, 1 ng expressed protein; 4, protein extracted from H3-size fruit; 5, protein extracted from H4-size fruit.

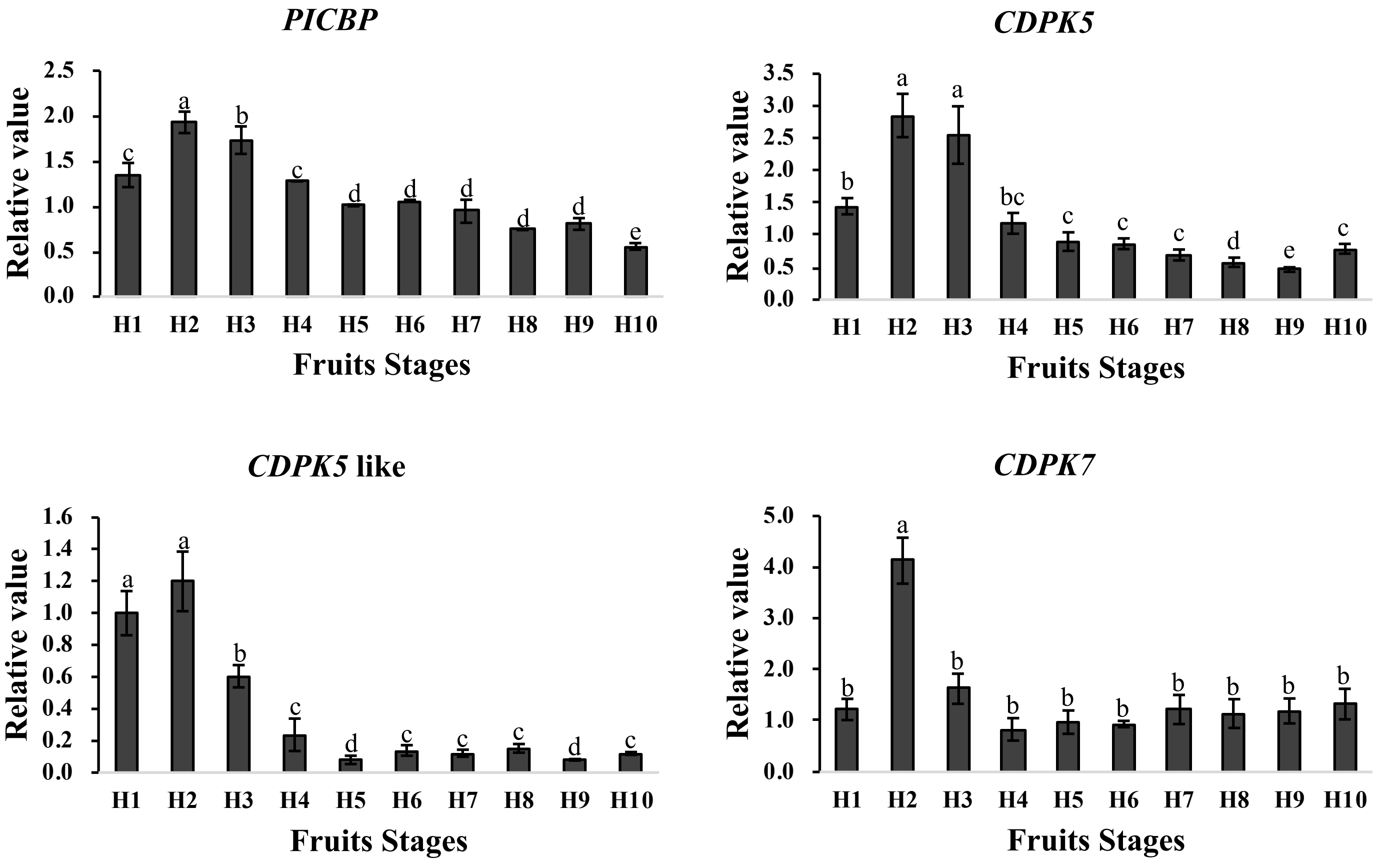
PICBP
Relative value
2.5
2.0
1.5
1.0
0.5
0.0
c a b c d d d d d e
H1 H2 H3 H4 H5 H6 H7 H8 H9 H10
Fruits Stages
CDPK5
Relative value
3.5
3.0
2.5
2.0
1.5
1.0
0.5
0.0
b a a bc c c c d e c
H1 H2 H3 H4 H5 H6 H7 H8 H9 H10
Fruits Stages
CDPK5 like
Relative value
1.6
1.4
1.2
1.0
0.8
0.6
0.4
0.2
0.0
a a b c d c c c d c
H1 H2 H3 H4 H5 H6 H7 H8 H9 H10
Fruits Stages
CDPK7
Relative value
5.0
4.0
3.0
2.0
1.0
0.0
b a b b b b b b b b
H1 H2 H3 H4 H5 H6 H7 H8 H9 H10
Fruits Stages

Fig. S6. Expression of *PICBP, CDPK5-like, CDPK5* and *CDPK7* genes.

The genes expressed in each size of fruit, with high expression observed in sample H2 (statistical results showed that these were at the middle stage of the initial cell stages, Fig. S1); different letters above the columns indicate significant differences at $P < 0.05$.

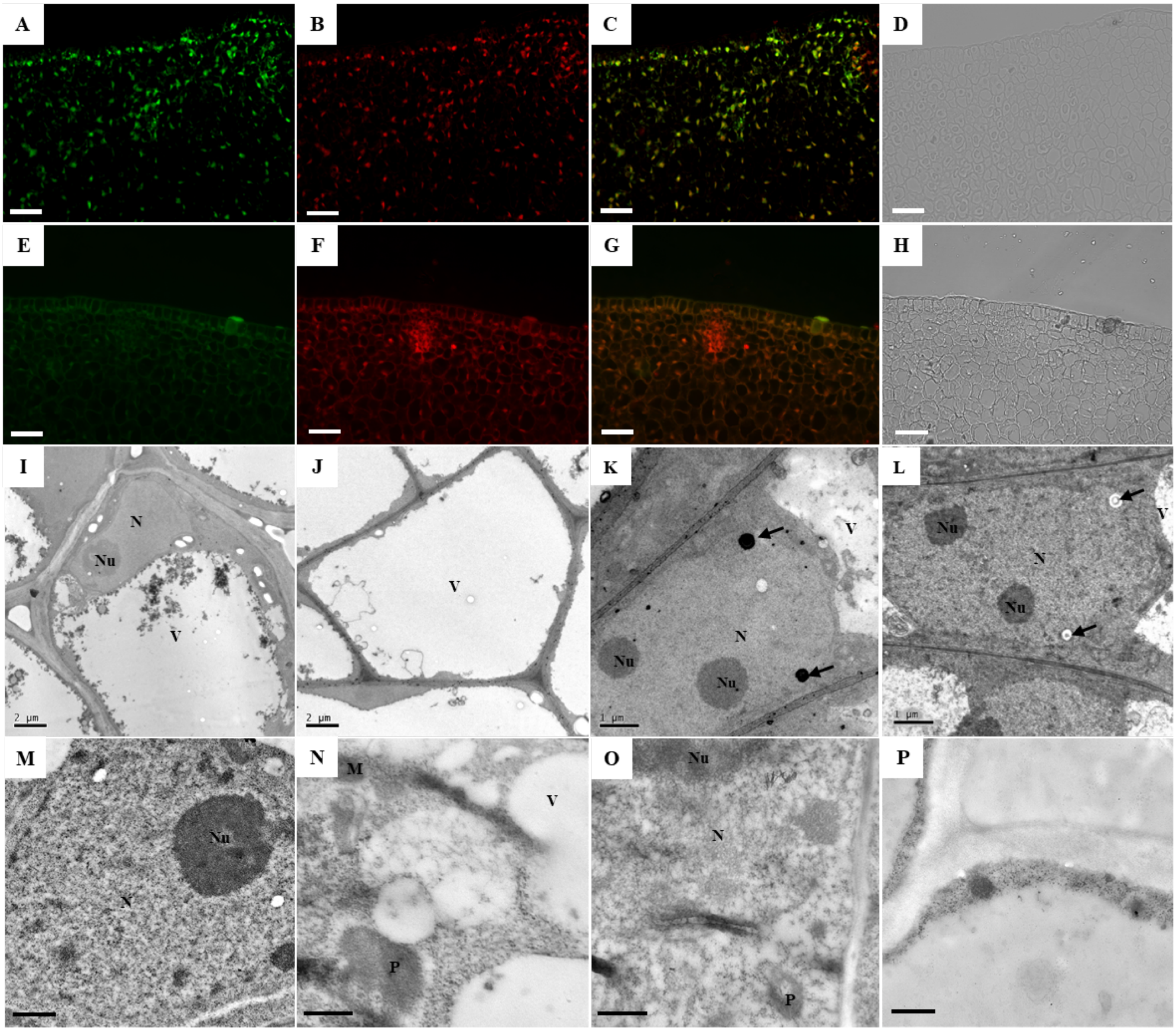
A
B
C
D
E
F
G
H
I
N
Nu
V
2 μm
J
V
2 μm
K
V
N
Nu
Nu
1 μm
L
Nu
V
N
Nu
1 μm
M
Nu
N
N
M
V
P
O
Nu
N
P
P

Fig. S7. Controls.

A-H. Control of the TUNEL assay; A-D show positive controls; E-H show negative control. Bar = 2 μm.

I, J. Control of the Ca^{2+} precipitates; showing living non-secretory cavity cells in *Citrus grandis* 'Tomentosa' fruits. A few calcium precipitates were only detected in the walls of the developing cell in young fruits (I) and in the walls of developed cells in mature fruits (J). Bar = 2 μm.

K, L. Calcium chelator EGTA-treated secretory cavity cells in *Citrus grandis* 'Tomentosa' fruits. (K) Numerous calcium precipitates (arrows) were observed in the PCD cells of the secretory cavity. (L) Calcium precipitates were cleared using the calcium chelator EGTA in the same nickel grid. Arrows indicate the same position. Bar = 1 μm.

M, N. Control images of the immunogold particles, showing the globular region. (M) shows control in which the antibody was replaced with preimmunization serum, (N) shows control in which the antibody was replaced with PBS. Bar = 500 nm.

O, P. Control images of the immunogold particles, showing living non-secretory cavity cells, in which no gold particles are present in the nuclei and nucleoli, mitochondria, plastids, and vesicles. Bar = 500 nm.

Table S1. The sequences of the gene specific primers.

Gene	Forward (5'-3')	Reserve(5'-3')
ACTIN	CAGTGGAGCCCTGCCCTTACC	GCGATCTCTGGCACCAACGGG
CgCaN(RT)	GCATGGAGAGAAGCAAAACC	CGACGAGTGTATGAGGCAGA
CgCaN	ATGGGAAATGCACTTAGGTTC	TCGGCCTTCGCGCCTGTC
CgCaN(P)	AAAAAGGATCCATGGGAAATGC ACTTAGG	AAAAAAAGCTTTCGGCCTTCG CGCCTGTC
PICBP	TGTGAAGTGGATGGCACACC	CTCTGTCCAAGCAGAAGCGA
CDPK5	AGAGCAACAACAACCGGACA	CGGGCTGTAGAACGGAAAA
CDPK5-like	TGACAAGCAATCGCATTCCG	GCGTCAGATTCCCTCCCTTT
CDPK7	GTGCCTCAGCTTGCTTACCT	TGCGTCAGTGGAGTTCTTCG

Note: *ACTIN* as the internal control is for quantitative real-time PCR analysis, *CgCaN(RT), PICBP*, *CDPK5*, *CDPK5-like* and *CDPK7* are for quantitative real-time PCR analysis, *CgCaN* is for gene clone, *CgCaN(P)* is for expression of *CgCaN* in the *in vitro E. coli*.
